# Gene expression in the mouse eye: an online resource for genetics using 103 strains of mice

**Published:** 2009-08-31

**Authors:** Eldon E. Geisert, Lu Lu, Natalie E. Freeman-Anderson, Justin P. Templeton, Mohamed Nassr, Xusheng Wang, Weikuan Gu, Yan Jiao, Robert W. Williams

**Affiliations:** 1 Department of Ophthalmology and Center for Vision Research, Memphis, TN; 2 Department of Anatomy and Neurobiology and Center for Integrative and Translational Genomics, Memphis, TN; 3 Department of Orthopedics, University of Tennessee Health Science Center, Memphis, TN

## Abstract

**Purpose:**

Individual differences in patterns of gene expression account for much of the diversity of ocular phenotypes and variation in disease risk. We examined the causes of expression differences, and in their linkage to sequence variants, functional differences, and ocular pathophysiology.

**Methods:**

mRNAs from young adult eyes were hybridized to oligomer microarrays (Affymetrix M430v2). Data were embedded in GeneNetwork with millions of single nucleotide polymorphisms, custom array annotation, and information on complementary cellular, functional, and behavioral traits. The data include male and female samples from 28 common strains, 68 BXD recombinant inbred lines, as well as several mutants and knockouts.

**Results:**

We provide a fully integrated resource to map, graph, analyze, and test causes and correlations of differences in gene expression in the eye. Covariance in mRNA expression can be used to infer gene function, extract signatures for different cells or tissues, to define molecular networks, and to map quantitative trait loci that produce expression differences. These data can also be used to connect disease phenotypes with sequence variants. We demonstrate that variation in rhodopsin expression efficiently predicts candidate genes for eight uncloned retinal diseases, including *WDR17* for the human *RP29* locus.

**Conclusions:**

The high level of strain variation in gene expression is a powerful tool that can be used to explore and test molecular networks underlying variation in structure, function, and disease susceptibility. The integration of these data into GeneNetwork provides users with a workbench to test linkages between sequence differences and eye structure and function.

## Introduction

Rapid progress in molecular biology, genomics, and bioinformatics combined with powerful mouse models have opened up many ways to study the genetics, development, function, and pathology of the eye and visual system [1-9]. An inevitable side effect of this relentless progress is that exploiting and integrating data are challenging. The purpose of this paper is to introduce a resource that binds together many data sets related to the genome, transcriptome, eye, and central visual system. Our goal is to improve the efficiency of making discoveries related to eye function and disease.

The foundation of this work is a massive gene expression data set generated using eyes from many of the most widely used strains of mice that are used in vision research and experimental genetics. A few examples of these interesting strains include:

• 129S1/SvImJ, one of a large family of related strains that have been used to generate almost all embryonic stem cells and knockout (KO) mice. This strain carries a mutation in the *Tyr* albino locus and in *Gnat2*—the achromatopsia gene.

• C3H/HeJ, a strain harboring the original *rd1* retinal degeneration mutation in the *Pde6b* gene.

• C57BL/6J, the single most widely used inbred strain of mouse. The genome of this strain has been extraordinarily well categorized. Many conventional and conditional gene KO lines are now generated and bred into this strain.

• DBA/2J, one of the oldest inbred strains of mice and a strain with mutations *Tyrp1* and *Gpnmb* that combine to produce a severe form of pigment-dispersion glaucoma [10,11].

• FVB/NJ, a common albino strain that also carries the *Pdeb6*-*rd1* rod degeneration mutation and that has been used to make the great majority of transgenic mouse lines.

These and many other strains differ greatly in their genomes. Any pair of strains will typically differ at 1–3 million known single nucleotide polymorphisms (SNPs), insertion-deletions-inversions, copy number variants (CNVs), and microsatellites [12]. A small fraction of these sequence variants—but still a high number—influence the development and function of the eye, and of course, susceptibility to disease. Animals from each line can be raised in tightly controlled environments. The combination of precisely defined genomes and precisely controlled environments provides an excellent foundation for experimental studies.

Why study the ocular transcriptome of over a hundred lines of mice? There are several answers. The first is that this type of survey makes it possible to define the level of normal genetic variation in expression [13]. This information is useful in answering simple, but important questions. For example, how much variation is there in expression of the three genes involved in age-related macular degeneration (ARMD)—complement factor H (*Cfh*), the retina-specific ATP-binding cassette transporter (*Abca4*), and apolipoprotein E (*Apoe*)? The answer is that there is greater than twofold variation in all three of these genes among normal isogenic lines of mice. This variation can be used as a tool in the same way that one would study KO and transgenic lines. The second reason is that it becomes possible to combine expression data across this large panel of diverse strains with well matched data on ocular and retinal histology and to generate genetic signatures of cells and tissue types. It is possible to determine sources of variation in the three ARMD genes at the cellular and molecular level. We demonstrate this powerful approach—a kind of statistical/genetic dissection of the eye—in the second part of the Results section using variation in rhodopsin expression to generate new candidate genes for uncloned human mutations that cause photoreceptor death and blindness. This powerful process can be extended to virtually all eye diseases for which there are one or more signature genes that are already known to contribute to disease onset or severity, the age-related macular degeneration genes being good examples.

Finally, the set of strains we have studied includes a large family of BXD recombinant inbred strains made by crossing two of the oldest and most widely used lines of mice: C57BL/6J (B) and DBA/2J (D). The eyes and retinas of this BXD family have been well studied for more than a decade, and we now possess extensive cytological and morphometric data on their eyes and retinas that can be studied with reference to differences in expression. With this large sample size, powerful statistical analysis is possible—for example, we can study correlations between numbers of retinal ganglion cells [14,15] or photoreceptors and the expression of specific genes.

The greatest utility of this BXD family is that it can be used to map the chromosomal positions of sequence variants that cause differences in gene expression, cell number, eye structure, and responses to retinal injury [15-17]. It is possible to advance from correlation to causation. For example, variation in the expression of over 500 transcripts in the eyes of BXD strains—many of which are related to the retinal pigment epithelium and pigmentation—can be traced back directly to the *Tyrp1* pigmentation-associated gene on chromosome (Chr) 4.

## Methods

### Animals

The HEIMED is based on data from 221 microarrays and 103 types of mice. Our goal was to obtain expression estimates for independent biologic samples from both sexes at approximately two months of age (young adult). The great majority of animals were obtained over a four-year period from colonies at University of Tennessee Health Science Center (Memphis, TN), and the Jackson Laboratory (Bar Harbor, ME). Mice were housed at 20 to 24 °C on a 14/10 h light/dark cycle in a specific pathogen-free (SPF) facility at the University of Tennessee. All animals were fed 5% fat Agway Prolab 3000 (Agway Inc., Syracuse, NY) mouse chow was provided ad libitum by water bottles. Eyes were obtained from DeltaGen Inc., (San Mateo, CA) and the KO stock was obtained from Ted Choi (Predictive Biology, Inc., San Diego, CA). *Rpe65* KO and *Nyx* mutant eyes were obtained from T. M. Redmond at the National Eye Institute (Bethesda, MD) and R. G. Gregg at the University of Louisville (Louisville, KY). Table 1 and Table 2 in the HEIMED information and metadata file provide key data on age, sex, and sources of all samples. Table 2 provides information on array quality and batch numbers. All experiments complied with the ARVO Statement for the Use of Animals in Ophthalmic and Vision Research.

**Table 1:** Knockout and mutant lines

**Gene**	**Chr@Mb**	**Expression level**	**QTL status**	**Age (Days)**	**n**	**Affymetrix probe set**	**Allele**
*Gabra1*	11@42	10.0	cisQTL	67–69	3	1421280_at	tm1Dgen
*Gabbr1*	17@37	10.9	none	16–22	5	1455021_at	tm1Dgen
*Gnb5*	9@75	13.0	none	22–25	3	1422208_a_at	tm1Dgen
*Gpr19*	6@135	9.6	cisQTL	68–70	2	1421756_a_at	tm1Dgen
*Clcn3*	8@64	12.4	none	67–69	3	1416610_a_at	tm1Dgen
*Rpe65*	3@160	12.6	cisQTL	57	2	1450197_at	tm1Tmr
*Nyx*	X@13	8.3	none	57	2	1446344_at	nob

This table lists the lines of mice that have had one gene knocked out. The table includes the gene symbol, its chromosomal location, QTL status in HEIMED, age of the mice, number of mice Affymetrix Probe set, and allele.

**Table 2:** Signature genes for cells, tissues, and systems of the mouse eye

**Index**	**Cell, tissue, or system**	**Primary gene signatures**	**Probe set IDs**	**Secondary signatures**
1	Sclera, firoblast cells	*Aebp1, Bgn, Fmod, Mxra8, Pcolce*	1422514_at, 1416405_at, 1415939_at, 1452330_a_at, 1448433_a_at	*Aldh2, Bgn, Col6a2, Enpp3 Igfbp6, Krt13, Lgals3, Mmp1, Mmp2, Mmp3, Scel, Serpinf1, Serping1, Sfn, Slc6a14, Timp1, Timp2, Timp3*
2	Sclera and cornea, fibroblasts (generic)	*Col1a1, Col1a2, Sparc*	1423669_at, 1450857_a_at, 1448392_at	
3	Cornea, epithelial cells, squamous cells	*Aldh3a1, Dsp, Krt12, Pkp1, Tjp3*	1418752_at, 1435494_s_at, 1419230_at, 1449586_at, 1417896_at	*Aqp3, Aqp5, Col5a2, Col6a3, Col12a1, Col17a1, Cs1, Dsg1, Dsc3, Gja1, Klf4, Klf5, Krt3, Krt17, Lgals3, Lypd3, Pax6, Perp, Tacstd2, Tkt, Upk1b*
4	Cornea, epithelium, basal cells (cuboidal, Bowman's layer associated)	*Col17a1, Sdc1, Tgfbi*	1418799_a_at, 1415944_at, 1415871_at	*Col7a1, Gsta4, Itga6, Ptgr1, Trp63*
5	Cornea and conjunctiva, limbal stem cells (and basal cells)	*Kit, Mki67, Trp63*	1452514_a_at, 1426817_at, 1418158_at	*Abcg2, Ereg, Nes, Pax6*
6	Cornea, stromal fibroblast keratocytes (neural crest derived)	*Col1a1, Col5a1, Col6a1, Col6a3, Kera, Lum*	1423669_at, 1416740_at, 1448590_at, 1424131_at, 1418063_at, 1423607_at	*Abcg2, Aim1, Aldo3, Aqp1, Col5a2, Cyp4v2, Dcn, Evpl, Fmod, Gsn, Gsta4, Gsto1, Krt5, Ogn, Ppl, Ptgds, Sfn, Sgce, Tacstd2, Tkt, Vim*
7	Cornea, endothelial cells (neural crest derived, Descemet's membrane associated)	*Col3a1, Mmp14, Slc4a11, Tgfbi, Wasf2*	1427883_a_at, 1448383_at, 1434867_at, 1448123_s_at, 1454673_at	*Aqp1, Apod, Car12, Cd274,Cd276, Col8a1, Col8a2, Dcn, Krt5, Krt12, Pitx2, Ptgs2, Svep1*
8	Conjunctiva, epithelium, squamous cells	*Krt7, Krt8*	1423952_a_at, 1423691_x_at	*Krt13, Krt14, Krt19*
9	Conjunctiva, goblet cells	*Muc5ac*	1430899_at	*Muc1, Muc4*
10	Anterior segment, leukocytes, macrophages, microglia	*Cd68, Ifitm1, Ly6a, Ly6e, Ptprc*	1449164_at, 1424254_at, 1417185_at, 1453304_s_at, 1422124_a_at	*Cd53, Hexb, Itgam, Lcp1, Lyve1, Tyrobp*
11	Anterior segment, dendritic cells	*Ctsc, Tlr2, Tlr3*	1416382_at, 1419132_at, 1422782_s_at	*Bcl10, Ctsc, Ifih1, Ifi202b, Tmem176b/Lr8, Il2rb*
12	Anterior segment, T regulatory cells	*Il2ra, Foxp3*	1420692_at, 1420765_a_at	*Cd4, Ctla4, Il2rb*
13	Anterior segment, cytotoxic T cells	*Cd8a, Ctsw, Gzbm*	1440164_x_at, 1422632_at, 1419060_at	*Cd28, Ctsw, Fasl, Prf1*
14	Anterior segment, NK natural killer cells	*Cd7, Klrb1c, Klrg1*	1419711_at, 1449570_at, 1420788_at	*Ctsw, Fcgr3, Klra17, Klrb1c, Klrd1, Klrk1, Ncam*
15	Anterior segment, immune suppression	*Cd55, Cd274*	1418762_at, 1419714_at	*Cd46, Cd59, Cd276, C3ib, Cgrp, Il1ra, Pomc1, Sst, Tgfb2, Tsp1, Vip*
16	Anterior segment, classical complement pathway	*C1qg, C1r, C1s, Ctss*	1449401_at, 1417009_at, 1424041_s_at, 1448591_at	*Bf, C1qa, C1qb, C2, C4b, Cd59, Ccl3, Ccl4, Cd14,Cd68, Coro1a, Csf1r, Ctss, Daf, Fcgf2b, Fcgr3, Serping1, Tyrobp*
17	Lymphatic endothelial cells	*Lyve1, Pdpn, Prox1*	1429379_at, 1419309_at, 1421336_at	*Flt4, Vegfc, Figf*
18	Extraocular muscles	*Atp2a1, Mylpf, Tnnc2, Tnni2, Tpm1*	1419312_at, 1448371_at, 1417464_at, 1416889_at, 1423049_a_at	*Acat1, Ache, Actc1, Actn3, Bgn, Cacng1, Chrna1, Ckm, Eno3, Lmod1, Mybpc2, Myh1, Myh4, Myh13, Pitx2, S100a1, Slc4a3, St8sia4, Tnni1, Tnnt2, Tnnt3, Tnt2, Tpm2, Ttn, Wsf1*
19	Aterrioles, smooth muscles	*Cnn2, Myl9, Tgln,*	1450981_at, 1452670_at, 1423505_at	*Acta2, Cnn2, Flna, Kcna3, Lmo2, Hdac9, Tpm2*
20	Arterioles, endothelial cells	*Efnb2, Efna1*	1419639_at, 1416895_at	*Adra2a, Kcna2, Nos3, Pecam1*
21	Vasculature, capillaries, endothelial cells	*Edg3, Pecam1, Vcam1*	1438658_a_at, 1421287_a_at, 1448162_at	*Ace, Ccl7, Ccl2, Ccl8, Cd31, Cd34, Cd105. Cd144, Cd248, Cd146, Cdh5, Cxcl1, Cxcl2, Cxcl3, Cxcl6, Edn1, Glycam1, Klf6 Nos3, Icam1, Il6, Ptprm, Plxdc1, Sele, Thbd, Vezf1*
22	Vasculature, venules, endothelial cells	*Nr2f2*	1416158_at	*Klf6*
23	Lens, capsule, equitorial region, epithelial cells	*Cryga, Crygf, Hspb1, Lenep*	1422652_at, 1429948_x_at, 1425964_x_at, 1422309_a_at	*Ablim, Cav1, Cltcl1, Cryba1, Crybb2, Crybb3, Crygb, Crydg, Crygs, Epb41l1, Epb49, Foxe3, Gsr, Gss, Maf, Picalm, Psip1, Prma7, Psma6, Psmb6, Psmb7, Psmb9, Psmd13, Sod1, Sorbs1, Sox1, Sptbn2, Tcp11*
24	Lens, fiber cells (primary, secondary), core and cortex	*Bfsp1, Bfsp2, Mip*	1450571_a_at, 1434463_at, 1421039_at	*Birc7, Lim2, Pacsin3*
25	Intrinsic eye muscles, sphincter and dilator	*Casq2, Mybpc1, Tnni1*	1422529_s_at, 1455645_at, 1450813_a_at	*Actg2, Ckmt2, Flnc Ppp1r12b, Myh2, Myh7, Tnni1, Tns1, Tpm3*
26	Melanocytes (general)	*Mlph, Myo5a, Rab27a*	1449896_at, 1419754_at, 1425285_a_at	*Edn3, Mltf, Notch2, Pldn*
27	Iris stromal pigment cells, iris pigment epithelium, myoepithelium	*Oca2, Slc45a2, Tyr*	1418211_at, 1451055_at, 1417717_a_at	*Clu, Mlph, Si*
28	Ciliary process	*Dct, Mlana, Tyrp1*	1418028_at, 1430635_at, 1415862_at	*Acta2, Adra2a, Arid5b, Gsta3, Hnmt, Hoxa10, Hoxa11, Krt8, Krt18, Mgll, Mlph, Pbx1, Si, Slc45a2, Srf*
29	Hyalocytes of the vitreous humor	*Has2, Has3*	1418678_at, 1420589_at	*Cd44, Cmas*
30	Trabecular meshwork, trabeculocytes	*A2m, Gsn, Mxra8, Myoc*	1434719_at, 1415812_at, 1452330_a_at, 1450468_at,	*Angptl7, Apod, Bgn, Ctgf, Foxc2, Gpnmb, Lgals8, Myoc, Pcolce, Scgb1a1, Sdc4, Sepp1, Serping1, Thbs1, Thbs2, Tnmd, Vim*
31	Choroid, choriocapillaris, choroidal endothelial cells	*Ptgds*	1449164_at	
32	Retina, retinal pigment epithelium	*Rpe65, Rdh5, Rgr Rrh, Ttr*	1418808_at, 1422832_at, 1450197_at, 1450280_a_at, 1459737_s_at	*Appbp1, Best1, Cst3, Ctsd, Kcnj13, Krt8, Krt18, Lrat, Mct3, Mct3l, Ptpgs, Rbp1, Slc16a8, Trf, Vmd2,*
33	Retina, Müller glial cells	*Slc1a3*	1443749_x_at	*Gfap, Glul, Rdh10, Slc6a1, Slc1a3, Vim*
34	Retina, rod photoreceptors	*Rho*	1425172_at	*Abca4, Cnga1, Gngt1, Sag, Pdc, Pde6g, Pde6b, Rom1*
35	Retina, cone photoreceptors, M type (green)	*Opn1mw*	1419723_at	*Arr3, Gnat2, Guca1a*
36	Retina, cones, UV/S type (blue)	*Opn1sw*	1449132_at	*Nr2e3, Thrb (developmental)*
37	Retina, horizontal cells	*Calb1*	1417504_at	*Lhx1, Prox1, Stx4a, Sept4,*
38	Retina, bipolar cells (generic)	*Vsx2*	1419628_at	*Bhlhb4, Cabp5, Gabrr2, Gnao1, Gng13, Hcn3, isl1*
39	Retina, OFF cone bipolar cells	*Tacr3, Vsx1, Glrb*	1437029_at, 1450487_at, 1422504_at	*Atp2b1, Gria2, Kcnip3, Neto1*
40	Retina, ON cone bipolar cells	*Gja7*	1447787_x_at	
41	Retina, rod bipolar cells	*Grm2*	1435607_at	*Glrb, Pcp2, Prkca*
42	Retina, amacrine cells, glycinergic	*Slc6a9*	1431812_a_at	*Cd44*
43	Retina, amacrine cells, AII rod type	*Dab1, Gria3*	1435578_s_at, 1438752_at	*Dab1, Gja9, Glra3, Ppp1r1b, Stx1a*
44	Retina, amacrine cells, A17 type (serotonin-accumulating, reciprocal)	*Slc6a4*	1417150_at	*Pah*
45	Retina, amacrine cells, A18 dopaminergic	*Th*	1420546_at	
46	Retina, amacrine cells, GABA-ergic	*Gad1, Gad2*	1416561_at, 1429589_at	*Cd15, Grid1, Grid2, Per1, Slc6a4*
47	Retina, amacrine cells, cholinergic starburst	*Chat*	1446681_at	*Kcnc1, Kcnc2*
48	Retina, retinal ganglion cells, generic	*Chrna6, Nrn1, Gap43, Thy1*	1450427_at, 1428393_at, 1423537_at, 1423135_at	*Bex1, Bex2, Calb2, Cerkl, Cplx1,Gja7, Map1a, Nef1, Nfl, Opn4, Pou4f1, Pou4f2, Pou4f3, Resp18, Stmn2, Slc17a6, Sncg, Vamp1, Vsnl1, Ywhah*
49	Retina, retinal ganglion cells, melanopsin photosensitive	*Opn4*	1421584_at	
50	Retina and optic nerve, astrocytes	*Gfap*	1426508_at	*Slc6a1, Vim, Slc1a3, Stat3*
51	Optic nerve, Oligodendrocytes	*Mog*	1448768_at	*Cldn11, Mag, Mbp, Mobp, Olig1, Plp1, Ugt8a*
52	Immediate-early response	*Egr1, Fos*	1417065_at, 1423100_at	*Jun, Ier2, Ier5*
53	Transcription	*Creb1, Rbbp6*	1428755_at, 1426487_a_at	
54	Histone modification	*Hdac2, Mbd1, Sirt1*	1445684_s_at, 1430837_a_at, 1418640_at	*Hdac3, Hdac7a, Hinfp, Mecp2, Rbbp7*
55	Cell cycle	*Cdc2a, Cdc20, Mki67*	1448314_at, 1439377_x_at, 1426817_at	*Birc5, Ccnb1, Cks2, Top2a*
56	Circadian rhythm	*Arntl, Clock, Per1, Per2*	1425099_a_at, 1418659_at, 1449851_at, 1417603_at	*Cry2, Dbp, Npas2*
57	miRNA processing	*Dicer1, Mtf1, Rnasen*	1460571_at, 1428979_at 1428656_at	*Pasha*
58	Apoptosis, unfolded protein response	*Apaf1, Cap12*	1452870_at, 1449297_at	
59	Anti-viral interferon induced response	*Ifit1, Oas1g, Oas2*	1450783_at, 1424775_at, 1425065_at	*Ifi1, Irf7, Mx1*
60	Oxidative phosphorylation, mitochondrial complex I, III, IV	*Cox8a, Ndufa11, Ucrc*	1448222_x_at, 1429708_at 1424364_a_at	*Nd5, Ndufb2, Ndufb10, Ndufs8,*

We have compiled a list of some of these signatures and present them in Table 2. As an example we can look at the cornea signature which uses *Aldh3a1* a corneal crystallin as a primary genetic tag and we can identify secondary signature genes.

### Inbred strains

Twenty-six lines were part of a mouse diversity panel (MDP) available from the Jackson Laboratory: 129S1/SvImJ, A/J, BALB/cJ, BALB/cByJ, BXSB/MpJ, C3H/HeJ, C57BL/6J, C57BLKS/J, CAST/EiJ, CBA/CaJ, CZECHII/EiJ, DBA/2J, FVB/NJ, KK/HlJ, LG/J, LP/J, MOLF/EiJ, NOD/LtJ, NZB/BlNJ, NZO/HlLtJ, NZW/LacJ, PANCEVO/EiJ, PWD/PhJ, PWK/PhJ, SJL/J, and WSB/EiJ. Of this set of strains, the most complete sequence data are available for C57BL/6J—about a 5X whole-genome shotgun sequence produced by the Mouse Genome Sequencing Consortium (2002) [18]. Three additional strains included in the MDP—129S1/SvImJ, A/J, and DBA/2J—were sequenced by Celera Genomics (Alameda, CA) with cumulative coverage of about 5X [18]. Fourteen strains were partially resequenced by Perlegen (Mountain View, CA) [19] using tiling arrays (129S1/SvImJ, A/J, AKR/J, BALB/cByJ, C3H/HeJ, CAST/EiJ, DBA/2J, FVB/NJ, KK/HlJ, MOLF/EiJ, NOD/LtJ, NZW/LacJ, PWD/PhJ, and WSB/EiJ). A key product of this first phase of sequencing is a set of 8.5 million common murine SNPs. All of these have been incorporated into a SNP Brower and are accessible with all HEIMED data in GeneNetwork. Any user can easily determine if there are known coding or noncoding sequence differences among common strains of mice for most genes of interest.

The MDP includes representatives of several different subspecies of *Mus musculus*, including *M. m. domesticus* (WSB/EiJ), *M. m. musculus* (CZECHII/EiJ), *M. m. castaneus* (CAST/EiJ), and *M. m. molossinus (MOLF/EiJ*). One strain belongs to a different species of *Mus*: *M. hortulanus* (PANCEVO/EiJ). The MDP also includes all eight parents of the Collaborative Cross [20-22] (129S1/SvImJ, A/J, C57BL/6J, CAST/EiJ, NOD/LtJ, NZO/HlLtJ, PWK/PhJ, and WSB/EiJ).

In addition to fully inbred strains, we have included a pair of reciprocal F1 hybrids made by crossing C57BL/6J and DBA/2J. These F1 hybrids—(C57BL/6J × DBA/2J)F1 and (DBA/2J × C57BL/6J)F1—are listed in the database using the common abbreviations B6D2F1 and D2B6F1, following the convention “strain of dam” × “strain of sire.” These F1 hybrids usually have expression levels intermediate between parents, but in principle, F1 hybrids can also be used to detect dominance and overdominance effects. Reciprocal F1 pairs can also be used to detect effects of imprinting on gene expression (e.g., *Gtl2*, probe set 1436713_s_at, and *Rian*, probe set 1427580_a_at).

### Knockout and mutant lines

We profiled whole eyes of five KO lines from Ted Choi that were generated by DeltaGen Inc. (San Mateo, CA; Table 1). Unlike all other lines in the HEIMED, these homozygous DeltaGen KOs are not isogenic, but are first generation backcross progeny (N1 progeny) of an F2 intercross between C57BL/6 and 129P2 (B6129P2F2N1). For this reason, we did not pool samples from these KO mice. Two of these KOs were in the GABA receptor family: the ionotropic alpha 1 receptor subunit, *Gabra1,* and the metabotropic beta 1 receptor subunit, *Gabbr1*. We profiled a DeltaGen *Clcn3* KO, a gene known to be associated with rod and cone photoreceptor degeneration [23]. The remaining two DeltaGen KOs inactivate *Gnb5* (*Gbeta5*), a gene essential in transducin deactivation [24], and *Gpr19*, a G protein-coupled receptor. We also studied an *Rpe65* KO line [25] on a (129X1/SvJ x 129S1/Sv)F1-Kitl^+^ genetic background and a BALB/cByJ strain with the spontaneous *nob* mutation in nyctalopin (*Nyx)* [26,27].

### BXD recombinant inbred strains

We studied 68 BXD recombinant inbred strains that segregate for three common coat and eye color mutations—dilute at *Myo5a*, brown at *Tyrp1*, and non-agouti at *Asip*. BXD1 through BXD32 were bred by Benjamin Taylor, Jackson Laboratory, Bar Harbor, ME starting in the 1970s [28], whereas BXD33 through BXD42 were bred by Taylor in the early 1990s [29]. BXD43 and higher were bred by L. Lu, J. Peirce, L. M. Silver, and R. W. Williams in the late 1990s and early 2000s using advanced intercross progeny [30]. All BXD strains are available from the Jackson Laboratory.

### Photoreceptor degeneration in inbred mice

Six strains of mice—C3H/HeJ, FVB/NJ, MOLF/EiJ, SJL/J, BXD24, and the *Clcn3* KO—have mutations that lead to rapid and early loss of rod photoreceptors. This loss of rods is nearly complete by one month of age and is often caused by the retinal degeneration 1 (*rd1*) allele in the rod cyclic-GMP phosphodiesterase 6-beta subunit gene, *Pde6b*. There is usually considerable “bystander” loss of cones in rod degeneration mutants, with half of all cones lost in the first month [31]. As expected from previous work [23], the loss of a functional *Clcn3* gene (chloride ion channel 3) also leads to rod and cone photoreceptor loss. BXD24/TyJ is the only BXD strain with retinal degeneration; it is caused by a spontaneous mutation that occurred between 1988 and 1992. (What we call BXD24 is JAX stock #000031, and is now referred to as BXD24b/TyJ.)

### Tissue and sample processing

Animals were killed by rapid cervical dislocation and the eyes were removed immediately. Optic nerves were trimmed at the orbit and most extraocular muscle was removed. Cleaned eyes were placed in RNA Later (Applied Biosystems/Ambion Foster City, CA) at room temperature. Both eyes from two to four animals of the same sex, age, and strain were stored in a single vial (four to eight eyes per pool). RNA STAT-60 Tel-Test Inc. (Friendswood, TX) was used to extract RNA from pooled eyes. All RNA samples were processed by one author (Y.J.). RNA was purified using procedures recommended by Affymetrix (Santa Clara, CA; sodium acetate in alcohol extraction), and 18S and 28S bands were examined on a 1% agarose gel. Only samples with a 260/280 ratio greater than 1.7 were accepted for further processing. We typically used a total of 8 µg of RNA for cDNA synthesis using a standard Eberwine T7 polymerase method [32] (Superscript II RT, Invitrogen/Life Technologies, Inc., Carlsbad, CA). The Affymetrix IVT labeling kit was used to generate labeled cRNA.

### Replication and sex balance

The samples were reasonably well balanced in terms of sample size and sex. Ninety-nine of 103 lines are represented by two or more pools of eyes, usually six eyes per pool-one male and one female pool. Sex differences in gene expression in the eye were minor (see Results), and data from males and females were combined to compute strain means and standard errors. Standard errors were corrected using the adjustment advocated by Gurland and Tripathi [33]. Two inbred strains were represented by a single male pool (BXD29 and A/J) or a single female pool (BXD69). Five KO and mutant lines (*Gpr19, Gabra1, Clcn3, Gabbr1, Nyx*) were represented by male samples only. Eyes from both sexes of SJL/J were inadvertently combined before hybridizing to the array.

### Affymetrix mouse genome 430 2.0 arrays and annotation

The M420 2.0 array consisted of roughly 1 million 25-nucleotide probes, grouped into 22 probes per probe set (11 match and 11 mismatch probes) that estimated the expression of approximately 39,000 transcripts and 19,459 genes (unique NCBI Entrez Gene IDs). The probe set sequences were selected late in 2002 using the Unigene Build 107 EST clusters [34]. We downloaded the latest annotation from the vendor (Mouse430_2.na28.annot.cvs) that was produced March 2, 2009. However, as will be described, we extensively reannotated the array content manually. The great majority of probes intentionally targeted the last coding exons and the 3′ end of transcripts—a part of the mRNA typically within 500 nt of the poly(A) tail. Many genes generated mRNAs with 3′ UTR length variants, and a large number of probe sets were designed by Affymetrix to specifically target both long and short 3′ UTR variants. Probe sets that targeted a region close to the primary polyadenylation site usually had higher signals than those probe sets that targeted secondary polyadenylation sites or regions of the transcript that were located in 5′ coding exons. The processing of the mRNA’s 3′ UTR was complex and different probe sets targeting different parts of the 3′ UTRs often gave very different estimates of expression. In general, probe sets that overlapped the last coding exon and the proximal part of the 3′ UTR provided the most representative signal that would match expectations of in situ hybridization results.

### Normalization

Affymetrix CEL files were processed using the Robust Multichip Array (RMA) protocol [35] with default settings. We then logged RMA values, computed Z scores for these RMA values for each array, multiplied Z scores by 2, and added an offset of 8 units to each value. The goal of this transformation was to produce a set of Z-like scores for each array that had a mean of 8, a variance of 4, and a standard deviation of 2. The advantage of this modified Z score was that a twofold difference in expression corresponded approximately to 1 unit. Finally, we computed the arithmetic mean of the values for the set of microarrays for each strain—usually two arrays and two pools of eyes per strain. (A single pair of technical replicates for strain NZW/LacJ were averaged before computing means of the two independent biologic samples.) Array data quality was evaluated using DataDesk 6.2 (DataDescription Inc., Ithaca, NY). Outlier array data sets were flagged by visual inspection in DataDesk, usually by means of an analysis of scatter plots, and more quantitatively by generating the correlation matrix of all arrays. In some cases, outliers were expected–for example, samples with retinal degeneration and samples from wild subspecies. However, when an array differed significantly from many other arrays and from its strain-matched companion, it was excluded from the study. We discarded approximately 10% of all arrays.

### Calibration

We calibrated expression using the Affymetrix spike-in controls. These 18 control probe sets targeted bacterial mRNAs that were added during sample preparation at four concentrations: 1.5, 5, 25, and 100 pM. (To find these probe sets, search the *ALL* search field in GeneNetwork using the string “*AFFX pM*.”) On the log2 scale, a value of 6 was equivalent to an mRNA concentration of approximately 0.4 pM, a value of 8 was equivalent to approximately 1.5 pM, 9.5 was equivalent to approximately 5 pM, 11.5 was equivalent to approximately 25 pM, 13.5 was equivalent to approximately 100 pM, and 15.5 was equivalent to an mRNA concentration of 400 pM or greater. This range can be converted to average numbers of mRNAs per cell in whole eye assuming that a value of 8–9 was equivalent to about 1–2 mRNA copy per cell [36]. For example, the expression of rhodopsin mRNA was 15 units in wild-type strains, equivalent to an average of 2^7^ to 2^8^ *Rho* mRNAs per cell. This is an underestimate of mRNAs per rod by a factor of about two. There were therefore likely to be about 500 *Rho* mRNAs per rod responsible for the replenishment of 80 rhodopsin proteins per second (70 million RHO proteins per rod with a molecular life span of 10 days [37].

### Batch structure

Arrays were processed in four batches. The complex and sequential normalization procedures used to combine batches are described in the GeneNetwork HEIMED metadata information file. The metadata standards available in this file are equivalent to Minimum Information About a Microarray Experiment (MIAME) and Gene Expression Omnibus (GEO) standards are presented in a conventional text format.

### Heritability index

We estimated the approximate fraction of variance in gene expression data due to genetic factors—a heritability index—using the simple equation of Hegmann and Poissedente [38]:

$$h^{2}=0.\text{5Va}/\left(0.\text{5Va}+\text{Ve}\right)$$,

where V_a_ was the additive genetic variance and V_e_ was the average environmental variance. The factor of 0.5 in this ratio was applied to adjust for the twofold increase of additive genetic variance among inbred strains relative to outbred populations. This decreased heritability estimates. However, this was counterbalanced to some extent because arrays were hybridized with sets of eyes from two to four cases, decreasing the environmental variance, V_e_. For these and other reasons, the heritability index should be used as an informal index of the strength of genetic control rather than as a firm determination. It is possible to determine the relation between the heritability index and the likelihood that gene loci can be mapped. Transcripts with heritability above 0.33 are often associated with one or more significant loci (e.g., *Polr1b*, see Results, Part 1).

### Data file availability

The final normalized HEIMED data set used in this paper and in GeneNetwork is available as strain means with error terms (data for 103 types of mice) and as a set of 221 individual arrays under the accession number GN207. The file names are as follows:

1. Geisert_EyeM430v2Sept08RMA103withSEM.txt (strain means and error of the mean)

2. Geisert_EyeM430v2Sept08RMA221cases.txt (individual pooled cases, male or female)

Due to the complex normalization procedure required to correct for batch effects we do not recommend using raw CEL files.

### Array annotation file availability

The custom annotation of the Affymetrix M430 2.0 array that is part of GeneNetwork is available by selecting “Mouse” as the species, and by selecting “Affy Mouse Genome 430 2.0 (GPL1261)” as the platform.

### Complementary web resources

Several complementary resources provide data on expression patterns in different tissues of the mouse, rat, and human eye using a variety of technologies and approaches. These sites can help define molecular signatures of different tissues in eye and the regulation of gene expression.

• The National Eye Institute Bank project [39] site provides a summary of expression data from multiple species with links back to GeneNetwork.

• 2. The Serial Analysis of Gene Expression (SAGE) database provides data on gene expression in the embryonic and postnatal mouse retina.

• 3. Differential gene expression in anatomic compartments of the human eye provides lists of transcripts with high expression in six ocular tissues.

• 4. The Retina Developmental Gene Expression site provides expression data for mouse retina at eight postnatal stages.

• 5. GeneNetwork contains data from a large eye transcriptome study of the adult rat based on 120 F2 intercross progeny using the Affymetrix RAE 230 v2.0 array [40]. All data are available by selecting Species=rat, Group=UIOWA SRxSHRSP F2, Type=Eye mRNA, and Database=UIOWA Eye mRNA RAE230v2 (Sep06) RMA.

• 6. RetNet, the Retinal Information Network, provides a summary of genes and loci causing inherited retinal diseases in humans.

## Results and Discussion

### Overview

This section is divided into four parts. In the first part, we explain how gene expression was measured and scaled, the level of variation in expression, and its heritability. We briefly review our custom annotation of the Affymetrix M430 2.0 microarray that makes data more useful to neuroscientists and vision researchers.

In part 2, we describe methods that take advantage of covariation and coexpression of transcripts to assemble networks. While the mRNA data are averages across many tissue types, the data set is sufficiently large that it is possible to statistically dissect sets of genes expressed in specific cells and tissue types. We show how to use expression signatures to build networks related to eye disease and generate a set of candidate genes for seven uncloned human retinitis pigmentosa loci.

In part 3, we introduce gene and quantitative trait locus (QTL) mapping methods. QTL mapping makes it possible to determine the directionality and causality of interactions among genes, their transcripts, and downstream targets [40,41].

In part 4, we describe methods to analyze networks of transcripts in combination with gene mapping. We provide an example of how sequence differences in the tyrosinase related protein 1 (*Tyrp1,* the brown locus*)* on chromosome (Chr) 4 controls the expression of large numbers of transcripts in the eyes of BXD strains.

Our intent in each of these four parts is to provide readers with enough information to carry out their own work and generate their own analyses. Figure legends include detailed instructions on how to exploit the data in combination with tools and resources that are part of GeneNetwork.

**Figure 1:**
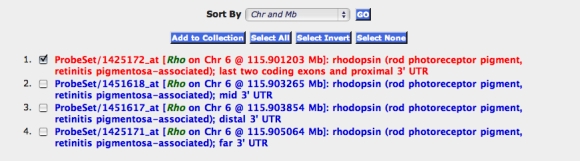
Extracting Data from the HEIMED. Step 1. Open the main website, GeneNetwork. Set up the Find Records pull-down menu fields to read: Choose Species=*Mouse*, Group=*BXD*, Type=*Eye mRNA*, Database=*Eye M430v2 (Sep08) RMA*. Step 2. Make these setting your default by clicking on the '**Set to Default**' button (bottom right of the window). Step 3. Enter the search term “rhodopsin” (quotes are not needed) in the ANY field and click on the '**Basic Search**' button. (Alternatively enter the search term “rhodopsin, rho” in the ALL field). Step 4. A Search Results window will open with a list of seven probe sets, four of which target different parts of the rhodopsin transcript. By default the probe sets are listed by their positional order from proximal Chr 1 to distal Chr Y. You can use the Sort By pull-down menu to reorder probe sets by average gene expression level, symbols, or by identifier numbers. Step 5. Click anywhere on the red text to generate a new window called the Trait Data and Analysis Form. The top of this window provides summary information on rhodopsin and this probe set; the middle section provides Analysis Tools; and the bottom section provides a set of editable boxes that contain the gene expression averages and error terms for all lines of mice starting with the B6D2F1 hybrids at the top and ending with the WSB/EiJ *Mus musculus domesticus* strain at the bottom (scroll to the bottom to see all of the common strains of mice).

To provide coherence across sections, many examples involve a common set of genes and their transcripts:

1. Rhodopsin, *Rho* (Affymetrix probe set 1425172_at; Figure 1), a marker for rod photoreceptors

2. Alpha 6 nicotinic receptor, *Chrna6* (1450426_at), a gene with high expression in retinal ganglion cells [42]

3. Choline acetyltransferase, *Chat* (1446681_at), a marker for a subset of amacrine cells

4. Glycoprotein nonmetastatic melanoma B, *Gpnmb* (1448303_at), a gene that contributes to glaucoma in DBA/2J

5. Tyrosinase-related protein 1, *Tyrp1* (1415862_at), a marker for the pigmented ocular tissues

6. Aldehyde dehydrogenase 3 alpha 1, *Aldh3a1* (1418752_at), a corneal marker gene

### Part 1: Measurement scale, variation, heritability, and probe annotation

#### Measurement scale

The average expression of all transcripts was 8 units with a standard deviation of 2. These measurements were on a log2 scale and each unit represented a twofold difference in mRNA concentration in the whole eye. Expression ranged from a low of 4.8 units (*Tcf15*, probe set 1420281_at) to a high of 15.5 (crystallin gamma C, *Crygc*, probe set 1422674_s_at). This range corresponded to a 1,660 fold difference (2^10.7^). However, the effective dynamic range was roughly half this value, on the order of 1 to 800. Expression of the six marker genes ranged from high values for those expressed in large populations of cells (15 units for *Rho*, 14 for *Aldh3a1*, 13 for *Tyrp1,* and 12 for *Gpnmb*) to intermediate values for genes expressed in smaller cell populations such as retinal ganglion cells and starburst amacrine cells (9.5 for *Chrna6* and 8 units for *Chat*).

#### Low expression values

**Figure 2:**
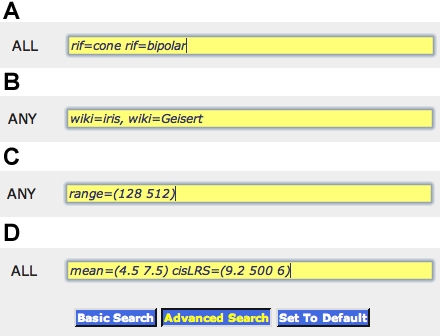
Advanced searching capabilities. Groups of genes, transcripts, and probe sets can be extracted from GeneNetwork using special query commands. To review the list of commands and their syntax, click on the '**Advanced Search**' button in GN, in the frame on the right side of the page. The search terms in the top panel **A** “rif=cone rif=bipolar,” when placed into the ALL field of GeneNetwork, will retrieve genes associated with cone bipolar cells, including *Atp2b1, Bsn, Gnao1, Gnb3, Gnb4, Gnb13, Grm7, Hcn1, Hcn2, Irx5, Kcnip3*, and *Vsx1*. This query exploits the constantly updated NCBI GeneRIF database that is integrated into GeneNetwork. The search terms in **B** will retrieve all probe sets that have been annotated by any user in the GeneWiki with either the word “iris” or the author’s name “geisert.” Panel **C** illustrates the use of the “range” command. This command is used to find transcripts that have different levels of variation across strains of mice. For example, the search string “*range=(128 512)*” will return mRNAs assays with greater than a 128 fold and less than 512 fold difference in expression across all 103 lines of mice. This is equivalent to a difference of 7 to 9 units (2^7^ and 2^9^). This search will return a list that includes *Cnga1 (*cyclic nucleotide gated channel alpha 1*), Gnat1* (rod alpha transducin), *Gsn* (gelsolin), *Mela* (melanoma antigen), *Nrl* (neural retina leucine zipper), *Pdc* (phosducin), *Pde6a, Pde6b, Pde6g* (three phosphodiesterases), *Rho* (rhodopsin), *Rp1* (retinitis pigmentosa 1), and *Sag* (S-antigen). Expression of *Sag*, for example, ranges from a low of 7.9 in the *Clcn3* knockout to a high of 15.4 in PANCEVO/EiJ, the colonial mound-building mouse species. Panel **D** illustrates a complex search that can be used to find probe sets with low expression but high genetic signal. This query finds all transcripts with expression levels between 4.5 and 7.5 that are also associated with strong evidence of a linkage peak (an LRS linkage scores >9.2 and <500) within 5 Mb of the parent gene, and where cisLRS is a shorthand to indicate that the quantitative trait locus (QTL) is near the location of the gene and has an LRS in a defined range. The cisLRS buffer parameter of 6 Mb in this query is equivalent to 0.5% of the mouse genome. Over 2,019 probe sets match these criteria, but there is a limit of 2,000 probe sets to view the complete results. In comparison, a total of 6375 probe sets—15% of the content of the array—match the query “mean=(4.5 7.5) LRS=(13.8 500).” These criteria are less restrictive and do not require transcripts to be controlled by their own gene locus (LRS versus cisLRS). However, they do require a higher LRS threshold equivalent to a LOD score of 3 (-logP=3, or p is approximately 0.001, where 1.0 LOD is roughly 4.6 LRS).

Many transcriptome studies exclude probe sets with low expression that are classified as absent or marginal [36]. Almost all would fall below 7 units on the log2 scale we have used. In contrast, we have included all probe sets and all values, even Affymetrix controls. Many probe sets with low values detect and reliably measure expression of the correct transcript. For example, expression of the calcium ion channel *Cacna2d1 (1440397_at*) varies more than twofold among strains—from 5.1 and 6.3 units (Figure 2D). This is well under the conventional detection threshold of the array and even below the background noise level of several genes that have been knocked out. However, by using gene mapping methods described in Part 3 it is possible to show that at least 70% of the variability in *Cacna2d1* expression is generated by polymorphisms that map precisely to the location of the *Cacna2d1* gene itself. This demonstrates that the arrays can achieve a reasonable signal-to-noise ratio even for some transcripts that are nominally declared to be absent.

#### Effects of mRNA dilution in complex tissues

While the overall concentration of mRNAs in the eye may be low, often averaging less than one mRNA per cell, concentrations within single cell types will often be much higher. In many array studies, dilution of message hampers analysis, but the HEIMED is sufficiently large that even modest signals can often be detected reliably. For example, *Chat* is only expressed in a small population of about 35,000 starburst amacrine cells [43]: 0.3% of the total retinal cell population. Nonetheless, expression of *Chat* in the whole eye is reasonably high and varies 2.2-fold from 7.6 to 8.7 (probe set 1446681_at). *Chat* expression covaries with other transcripts known to be expressed selectively in starburst amacrine cells, including *Kcnc2* (*r*=0.69), [44] and *Slc2a3* (*Glut3, r*=0.69), [45].

#### Variation in array signal across strains

**Figure 3:**
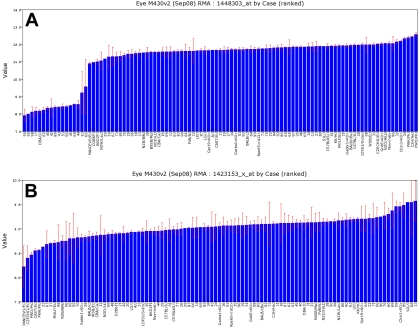
Variation in gene expression. These bar charts summarize data for *Gpnmb* and *Chf* across 103 strains, with strain names or numbers along the x-axis (BXD1 is abbreviated 01). The y-axis indicates expression on a log2 scale. Bars are standard errors of the mean. **A:** Variation in gene expression of the glaucoma gene *Gpnmb* (probe set 1448303_at) indicates that fifteen BXD strains have low expression and can be used as models for glaucoma, retinal ganglion cells degeneration, and defects of innate immunity [80]. The 25-fold decrease in expression of *Gpnmb* in DBA/2J and 15 of the new BXD strains (left side) is caused by a mutation that introduces a premature stop codon in the middle of exon 4 (R150X, CGA to TGA, Chr 6 nucleotide 48.974971 Mb) [10,80]. This mutation eliminates the target region of the transcript and enhances nonsense-mediated RNA decay of the truncated transcript. This variation in expression maps as a strong cis QTL. **B:** There is a 2.6 fold range of expression of complement factor H (*Cfh*), probe set 1423153_x_at) this is determined by generating bar charts of strain variation in gene expression. How to generate bar charts of strain variation in gene expression. Step 1. Work through the steps described in Figure 1 using *Gpnmb* as the search term in Step 1. Step 2. Once you have opened the Trait Data and Analysis Form shown in Figure 4 below, select the '**Basic Statistics**' button.

There is substantial variation in gene expression among strains of mice (Figure 2C, Figure 3). Nearly half of all probe sets have more than a twofold range; 12% have more than a fourfold range; and 4% have more than a eightfold range. Rhodopsin is an example of a transcript with an extreme 300-fold difference (Figure 1), for the simple reason that several strains have no photoreceptors. Eliminating those strains with photoreceptor mutations reduces the range to 1.7-fold. The interquartile range—the difference in expression between strains at the 25% and 75% levels—is a conservative and robust way to estimate variation that is unaffected by outliers. For example, even with the retention of the six-outlier *rd* strains, the interquartile range for rhodopsin is only 1.16-fold.

Expression differences need to be interpreted cautiously. Sequence variants among strains often produce variation in hybridization efficiency that can mimic a true expression difference. Strain variation in the length of mRNAs, in particular, longer and shorter 3′ untranslated regions (UTR) can also change signal intensity. Bioinformatic methods can test the likelihood that differences are authentic, but the best test is quantitative PCR with primers that amplify constitutive coding exons.

#### Sex differences in gene expression in the eye

Sex differences in gene expression in the eye are modest. Only 141 probe sets (0.3%) are differentially expressed at a false discovery rate of 0.05 using a powerful paired *t*-test with 93 male–female contrasts (Table 1, Appendix 1). Not surprisingly, transcripts with the largest sex differences are located on the X and Y chromosomes. These differences are generic dosage effects that can be detected in almost all tissues. Examples include *Ddx3Y, Jarid1d,* and *Eif2s3y* on the Y chromosome and *Xist, Jarid1c*, and *Utx* on the X chromosome. The imprinted gene *H19* on Chr 7 is the most differentially expressed autosomal gene (50% higher in females, 9.79±0.04 versus 9.21±0.06). Genes with sex differences and high expression in the eye include parvalbumin (*Pvalb*, 24% higher in males), a gene regarded as a marker of AII amacrine cells [46] and glutathione peroxidase 3 (*Gpx3*, 20% higher in males), a gene associated with macular degeneration. In general, however, sex differences were so small that we treated male and female pools as strain replicates without a correction for sex. This allowed us to provide error terms for expression estimate for the great majority of strains and to estimate the approximate heritability of expression variation.

#### Heritability of variation in expression

**Figure 4:**
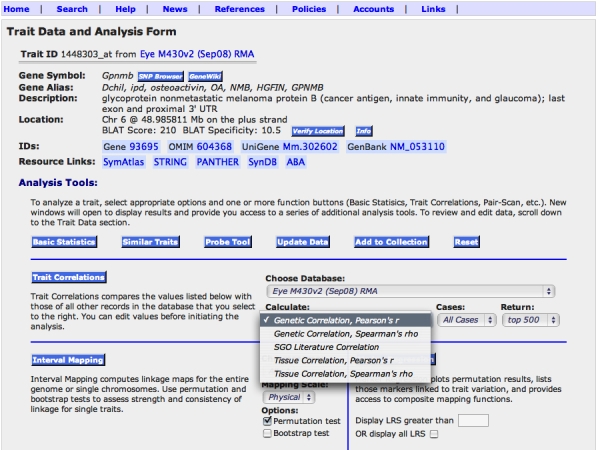
Analysis tools available from the Trait Data and Analysis Form. These functions are used to study data on variation and covariation of gene expression. The 11 function buttons do the following: 1. '**SNP Browser**' lists known single nucleotide polymorphisms (SNPs) in *Gpnmb* among all strains for which data are available. 2. '**GeneWiki**' provides a tool for any user to annotate any gene and leave references and notes on their expression. 3. Verify Location function is used to retrieve the precise genomic location of probe from the UCSC Genome Browser. 4. The '**Info**' button explains the Verify Locations function above. 5. The '**Basic Statistics**' function generates simple univariate statistics, including the heritability index. Selecting this function generated the bar charts reproduced in Figure 3. 6.The '**Similar Traits**' function finds expression data for *Gpnmb* in other tissues such as the cerebellum, striatum, hippocampus, neocortex, kidney, and liver. 7. The '**Probe Tool**' provides access to the low level probe data (CEL file level data). 8. '**Add to Collection**' moves *Gpnmb* expression data for the 71 BXD family members into a BXD Trait Collection window (similar to a shopping cart) that can include over 100 other expression or trait data for these particular strains. 9. The '**Reset**' function resets all values to their original values and settings (values in the Trait Data and Analysis form can be edited by the user during an analysis). 10. The '**Trait Correlation**' function finds the top data sets with matched expression patterns using complementary methods shown in the pull-down menu: Genetic Correlations (strain correlations generated using the HEIMED data itself), Semantic Gene Organizer (SGO) Literature Correlations, and Tissue Correlations. By default this function will return the top 500 covariates, but this can be changed from the top 100 to the top 2000. 11. Interval Mapping tests whether the variation in expression of a transcript in the BXD strains is strongly linked to sequence differences in a particular part of the genome. Using this function for *Gpnmb* generates a map that shows a strong QTL (LRS of 33.1) on Chr 6 at the precise location of the *Gpnmb* gene itself.

**Figure 5:**
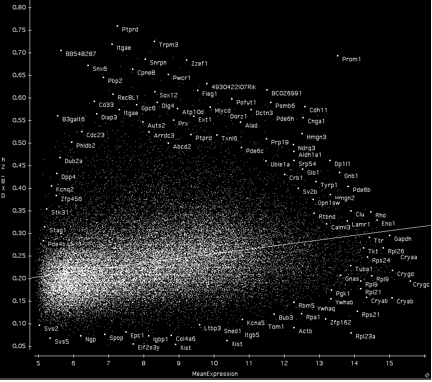
Heritability and gene expression level. There is a trend among transcripts with the highest heritability (>0.35) to have intermediate expression (seven to 11 units). Transcripts with low heritability and high expression tend to be housekeeping genes, including probe sets for ribosomal transcripts (lower right corner, e.g., *Rpl23a*, *Rps21*, *Rpl9*) and many of the crystallin transcripts (*Cryab*, *Crygc*, and *Crygd*). Despite the exclusion of all strains with retinal degeneration, including BXD24, a large number of retinal and RPE transcripts (*Rho*, *Pde6b*, *Opn1sw*, *Cnga1*, *Rtbnd*, *Prom1*, *Pde6c*, *Dp1l1, Reep1*, *Clu*, and *Tyrp1*) have comparatively high heritability (>0.33). The low heritability of transcripts such as *Xist* and *Eif2s3y* (bottom middle) is due to the study design that does not account for within-strain differences in a small number of genes on the X and Y chromosomes with strong sex-specific expression (see section on sex differences).

Heritability is the fraction of variation caused by genetic effects. In our data set the variation in expression was due to genetic differences between the strain means, environmental effects, and technical error. We computed an index of heritability using data from the BXD family and their parental strains, C57BL/6J and DBA/2J. This method provided better insight into the likelihood that mapping QTLs that influence gene expression will be successful. The values ranged from a high of about 0.75 (the majority of variance associated with strain differences) to a low of about 0.05. These values should be used as an index of heritability rather than as true estimates because we used pooled samples to maximize genetic effects and minimize environmental variance. However, to provide a somewhat more realistic estimate of heritability, as would be calculated using individuals from an F2 population, we applied the correction of Hegmann and Possidente [38]. The adjusted mean heritability for all probe sets was 0.24±0.07 (±standard deviation). These adjusted values are listed in each of the basic statistics pages for Affymetrix probe sets. For example, the heritability index of *Tyrp1* is 0.44, that of *Rho* and *Chrna6* are 0.28–0.30, whereas those of *Chat* and *Aldh3a1* are about 0.25. If transcripts have values of 0.33 to 0.34, it will be possible to map one or more QTLs that modulate expression in about 25% of cases. For example, the heritability index for *Gpnmb* shown in Figure 3 and Figure 4 is 0.48, and is associated with a strong QTL. Similarly, *Tryp1,* with a heritability index of 0.44 using probe set 1415862_at, is associated with a QTL superimposed over the gene itself. In contrast, *Rho, Chat*, and *Aldh3a1*, with values under 0.33, are not associated with strong QTLs. However, *Chrna6* is somewhat exceptional; although it has a heritability of only 0.28, expression of this transcript is modulated by a strong QTL on proximal Chr 8 with an LRS score of 25.2, equivalent to a LOD score of 5.5. We expected to find a strong correlation between heritability and expression level (Figure 5), but we observed transcripts with the highest heritabilities (>0.6) and strongest QTL mapping results tended to have low to moderate expression.

#### Custom array annotation

The value of microarray data sets is a direct function of the quality of the array annotation. Unfortunately, annotation for the Affymetrix M430 2.0 array (version 28 of March 2009) is still incomplete, sometimes incorrect, and does not provide information to select among multiple probe sets targeting the same transcript. This is critical because over 15% of probe sets target introns, rare EST, or antisense sequence. These probe sets should generally not be used to measure expression of protein-coding mRNAs. The standard annotation for families of genes is also particularly problematic. For example, 4 of 31 probe sets that target crucial crystallin transcripts, incorrectly target introns and one targets an antisense sequence.

As part of the development of the HEIMED, we manually annotated probe sets by BLAT sequence alignment to the mouse genome and transcriptome. Approximately 14,000 probe sets that had comparatively high expression in eye and central nervous system (CNS) had been curated by one of the authors (R.W.W.) and often had information on regions of transcripts that were targeted by probe sets (Figure 1). For other probe sets we generated closely matched data with somewhat less precision using computational methods (BLAT analysis combined with annotated genome sequence). We also occasionally added short descriptive tags to genes associated with eye disease and vision to make it easier to navigate the data set. For example, the annotation for *Cerkl* read, “neuronal survival and apoptosis-related, retinal ganglion cell expressed, retinitis pigmentosa 26.”

#### How to choose among alternative probe sets

Annotation is critical in deciding which of several probe sets will give better estimates of expression. In general, we recommend those probe sets with high expression that target the last coding exons or the proximal part of the 3′ UTR. As shown in Figure 1, we added information of this type for most probe sets and transcripts. In the case of the four rhodopsin probe sets, the first one targeted the last two coding exons, whereas the other probe sets targeted the mid, distal, and far distal parts of the 3′ UTR. These four probe sets can be sorted by their average expression level using the *Sort By* function described in the legend to Figure 4. In the case of *Rho*, the probe set that targeted the last two coding exons and the proximal 3′ UTR had the highest expression and was an optimal choice for an analysis of variation in rhodopsin expression (Figure 1, red font). In general, the Affymetrix M430 2.0 probe sets intentionally target the 3′ end of transcripts and provided an averaged estimate across multiple isoforms. In other words, this is not a suitable platform to study expression of specific splice variants. To do this would require data generated either using an exon array (Affymetrix Exon 1.0 ST) or deep sequencing of mRNA-derived libraries [47].

### Part 2: Correlations among transcripts and traits

In this section, we summarize sources of covariation among transcripts and phenotypes such as cell number in the eye. We also provide two relatively complete examples of how signature genes and their covariates can be exploited to define molecular and phenotype networks. In the first case, we use a single gene, rhodopsin, as starting material. In the second case, we use a set of three genes that have been used as markers of retinal ganglion cells.

While HEIMED data can be used to compare pairs of strains or sets of genotypes, it is usually more useful to compute correlations across the complete data set of 103 strains. With just over 45,000 probe sets this can produce a matrix of up to 1.017 billion correlations, each computed using data across the full panel of strains. The strength and polarity of correlations can be used to test specific hypotheses, to assemble large interaction networks, or for exploratory analysis.

#### Strain genetic correlations

**Figure 6:**
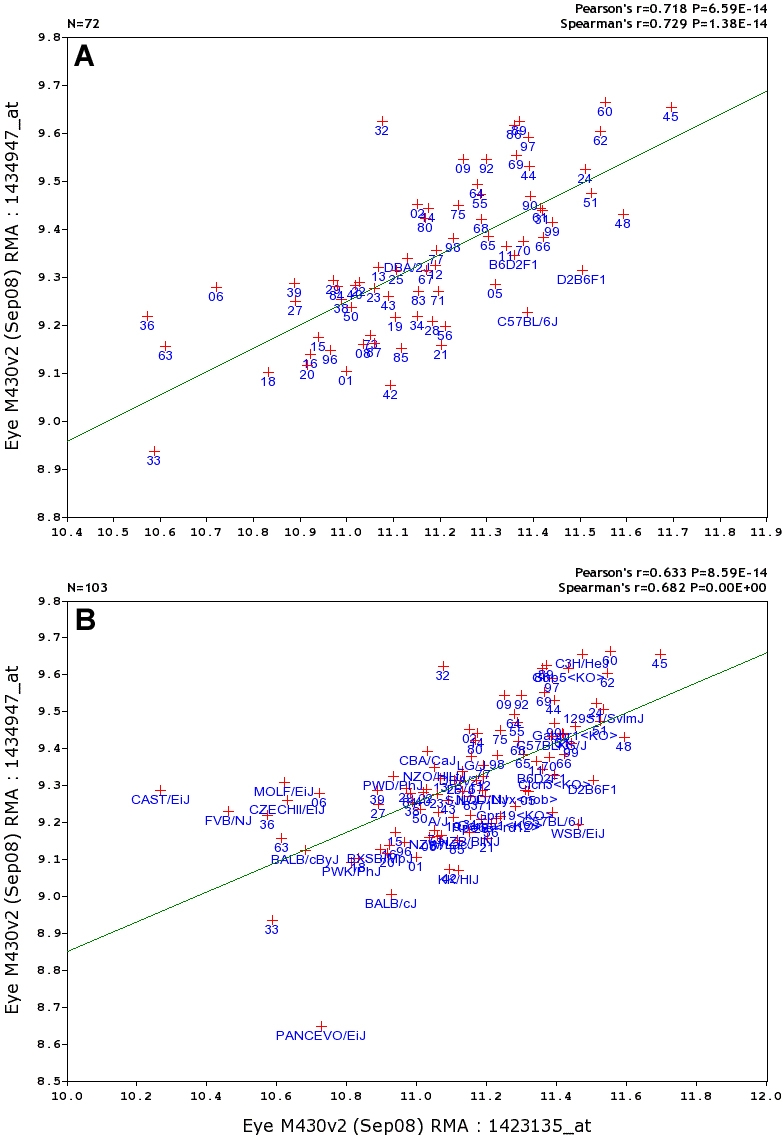
Correlation scatter plots for retinal ganglion cell markers. Pearson and Spearman correlations are listed in the top right corner, along with p values. **A** provides the correlation using only the BXD family strains (n=72), whereas **B** provides data for the full set of 103 types of mice. How to compute correlations between two genes, such as *Thy1* and *Kif3c*: Step 1. Link to GeneNetwork and search for probe sets 1423135_at and 1434947_at in the ANY field (or search for *Thy1* and *Kif3c* rather than the specific probe set). Step 2. Click the check boxes to the left of each entry and click the '**Add to Collection**' button. GeneNetwork will place probe sets in a BXD Trait Collection window. You can add many of other traits to this window, but they must all be traits associated with the BXD group of mouse strains. Step 3. Select the check boxes again in the BXD Trait Collection window, and then click the '**Correlation Matrix**' button. This computes both Pearson and Spearman correlations and places them in a 2×2 correlation matrix. (You can make a correlation matrix with up to 100 entries.) All of the values in this 2×2 matrix are linked to scatter plots. Step 4. Click on the lower-left square (it should read 0.718 n=72). This will open a scatter plot of the coexpression of the two probe sets, panel **A**. A simple alternative method will give you the plot shown in panel **B**. Search for the *Thy1* as in step 1, then click on the entry text itself rather than the check box. This will open the Trait Data and Analysis Form for *Thy1* (see the *Gpnmb* example in Figure 4). Find the button labeled '**Trait Correlations**' and select it, leaving the other settings (Choose Database, Calculate, Case, and Return) in their default settings. A Correlation Table will automatically open with a list of the top 500 correlates of *Thy1* based on the variation across all 103 types of mice. Item 9 on this list of 500 transcripts is the *Kif3c* probe set 1434947_at. Finally, click on the blue correlation value in the *Kif3c* row (row 9) to regenerate panel **B**. Review each column of data and note that the list of 500 can be resorted using the small up and down arrowheads at the top of each column.

We refer to correlations computed using strain means as genetic correlations although they are actually produced by a mixture of several sources of variation, including genetic factors, technical error and noise, and uncontrolled environmental variation. Each correlation was linked with a scatterplot, such as that between two transcripts associated with retinal ganglion cells: *Thy1* and *Kif3c* (Figure 6). By default these scatter plots were generated using data for all 103 lines (Figure 6B), but scatter plots can also be computed for just the BXD family of 72 strains (Figure 6A) or just for the diversity panel consisting of 35 strains. Each point represented a single strain mean. In some cases, it helps to remove outliers, such as PANCEVO/EiJ in Figure 6B. This can be done in any Trait Data and Analysis page in GeneNetwork by manually deleting individual strain data. In the case of *Thy1*, this involved replacing the value of 10.728 for PANCEVO/EiJ with the value “x” in the Trait Data and Analysis data field and recomputing the list of top 500 covariates.

Correlations help define functional units within cell types or across cell types and tissue boundaries. At some level of measurement, the balance and stoichiometry of molecular interactions are important and provide insight into functional modules. A tight correlation between transcripts with high expression and high heritability across a large number of different strains will have a biologic cause. The strong molecular footprint left by rod photoreceptors on the expression of *Rho* and hundreds of other transcripts is a good example. A subtle example is the tight correlation between pairs of photoreceptor and bipolar cell transcripts. The ON-bipolar cell signature gene, *Gnao1* [48,49] (probe set 1421152_a_at), covaries with *Sv2a*, a key presynaptic gene expressed in cones [50]. The ganglion cell maker, *Chrna6* covaries well with the AII amacrine maker *Gria4*. In this case, correlations are equal to or greater than 0.8 using either Pearson’s r or Spearman *rho*. These types of correlations across cell types and tissues can provide important functional insight that can only be detected when expression of groups of cells are studied together.

#### Triangulating gene function using literature and tissue correlations

Two independent types of correlations—literature correlations and tissue correlations—complement the genetic correlations (Figure 6). Literature correlations were generated using the Semantic Gene Organizer [51]. These values are based on the similarity of sets of terms associated with pairs of genes [52]. Roughly 5% of literature correlations have values above 0.6. In the case of *Thy1* and *Kif3c,* the literature correlation is 0.56.

Tissue correlations provide a third independent method of computing correlations. These values estimate coexpression of genes across 25 different tissues and organs (e.g., lung, spleen, liver, brain, testis, eye). Tissue correlations and associated scatter plots of expression variation between two genes are useful in evaluating the specificity of expression in eye or other tissues. As expected, *Thy1* has its highest expression in the thymus, whereas *Kif3c* has its highest expression in CNS and muscle. Correlations of expression of these two genes across 25 tissues are approximately 0.64 using both Spearman and Pearson methods.

These three types of gene-gene correlations—genetic, literature, and tissue—can be used to obtain complementary perspectives on network membership. This is particularly useful when a gene has not been well studied. The methods can sometimes be used to triangulate gene function in the same way that peptide sequence is often used to predict protein function and location.

#### Correlations with classic phenotypes

Correlations can be extended to a wide variety of other types of traits measured in many of the same strains. We have assembled a database with hundreds of phenotypes for the BXD strains, including data on eye and lens weight, retinal area [16,53,54], photoreceptor and retinal ganglion cell number [15,55], cell populations in the dorsal lateral geniculate nucleus [17], and even data on plasticity in visual cortex of BXD strains following monocular deprivation [9]. Any of these traits can be correlated with eye expression data. For example, difference in the number of retinal ganglion cells covaries with *Fbxl20*, *Med1,* and *Cacs3*. These genes are located in a region of Chr 11 that is known to control the proliferation of this cell population. By combining the correlation with other published BXD data sets [14,15], we can show that the correlation with *Fbxl20* is a genetic and developmental imprint rather than the result of adult function of this gene in ganglion cells, a topic to which we return at the end of this section.

#### Molecular signatures of tissues and cells

Shared patterns of expression can help define genes with specialized roles in specific cell, tissue types, or even heterogeneous systems that involve multiple tissues such as ocular innate immunity, neuromodulation, or intraocular pressure control. To help parse the expression data we compiled an extensive table of several known signature genes and matched probe sets for many different cell and tissue types, and a few examples of systems that involve multiple cell types. These signatures can be used for exploratory analysis of other transcripts that may be expressed in, or associated with, the same types of cells or systems (Table 2).

These lists in Table 2 should be considered provisional for several reasons. First and foremost is the issue of specificity. Many genes reported to be signatures for specific cell types actually have widespread expression in the eye. For example, calbindin-28 kDa (*Calb2*) is a marker for both horizontal cells and a subpopulation of retinal ganglion cells (e.g., [56]). Second, even with a large sample size, correlations are relatively noisy, and given the huge number of correlations that can be screened in GeneNetwork, the false discovery rate will be high with r values of less than 0.5.

There are several good ways to empirically evaluate a list of gene covariates. The easiest way is to compare the three different types of correlations—genetic, literature, and tissue—and look for moderate to high values (r or rho > 0.4) in all three (Figure 6).

#### Gene ontology analysis

**Figure 7:**
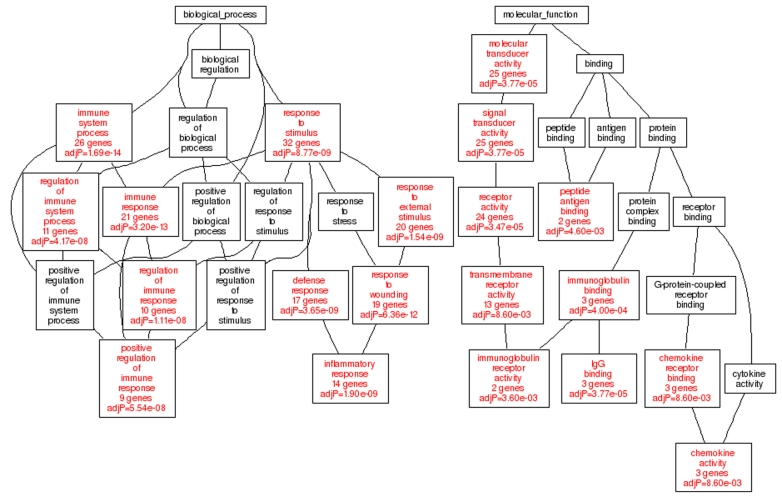
Gene ontology for innate immunity. These data reveal that correlates of *Ptprc* (1422124_a_at) are related to the biology of innate immunity. How to generate WebGestalt’s Geneset Ontologies through GeneNetwork. For the purpose of identifying the Geneset Ontology of the Innate Immunity signature network, a correlation of *Ptprc* (1422124_a_at), a leukocyte, microglial marker gene was used. As shown in Figure 4, a Trait Correlation was run and set to return the top 100 genes. At the top of the resulting Correlation Table, the '**Gene Ontology**' button was selected which sends the 100 transcripts in this Correlation Table to WebGestalt for GOTree analysis. When complete, there are three options: Directed Acyclic Graph (DAG), Export TSV, or Export DAG. For this figure the DAG was chosen and the biological_process and molecular_function listings were displayed.

The second method to evaluate the biologic significance of sets of correlations is to perform a gene ontology (GO) analysis of the covariates using WebGestalt [57]. WebGesalt is a powerful online GO analysis resource that includes a custom interface with GeneNetwork that makes it particularly easy to carry out an analysis. The steps required to generate a GO analysis starting with a list of from 100 to 2,000 genes or transcripts is described more fully in Figure 7.

**Figure 8:**
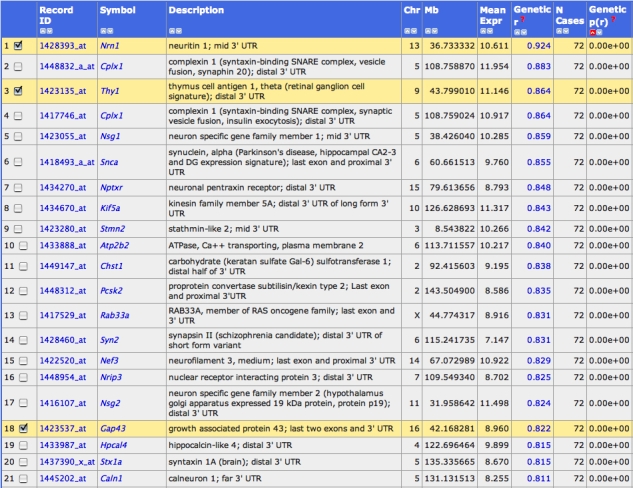
A list of genes associated with retinal ganglion cells. Rows 1, 3, and 18 list three ganglion cell signature genes used as bait with which to trap new candidate genes. How to generate a synthetic trait from three or more transcripts: Step 1. Select a set of transcripts or other traits (even classic phenotype will work) and add them to the Trait Collection as described in the legend to Figure 6, steps 1 and 2. For example, add the transcripts for *Thy1*, *Nrn1*, and *Gap43* (probe sets 1423135_at, 1428393_at, 1423537_at). Step 2. Select the check boxes of the probe sets in the Trait Collection window and then click the '**Correlation Matrix**' button. A new window will open. Scroll down to the section labeled PCA Traits. One or more synthetic traits will be listed here. PC01 is the synthetic trait that shares the most in common with the set of traits that you submitted for analysis. Step 3. Click on the blue text of PCA Trait PC01. This will open a Trait Data and Analysis page that can now be used for various functions, including mapping and correlation analysis. Step 4: To find other transcripts that share features with the PC01 trait constructed using *Thy1*, *Nrn1*, and *Gap43*, scroll to the '**Traits Correlations**' section of the Trait Data and Analysis page. Before clicking the Trait Correlation button change the Choose Database pull-down menu to read Eye *M430v2* (Sep08) RMA data.

The first cause—cell population variation—contributes to the strong correlations between rhodopsin and many other photoreceptor-associated transcripts. These correlations are produced mainly by the extreme differences between the retinal degeneration *rd* mutants and the *rd* wildtype lines. It would normally be difficult to determine if population structure had a role in covariation, but in the case of photoreceptors and retinal ganglion cells, we have good estimates of numbers of cells in many strains [15,55]. It is therefore possible to correlate cell population with expression levels as outlined in Figures 6 and Figure 8, but starting with the BXD phenotype database in GeneNetwork and searching for either the term “photoreceptor” or “retinal ganglion cell” in the ALL field.

#### Genetic covariance of to define new candidate RP genes

Strain differences in rhodopsin expression (Figure 1, probe set 1425172_at) were used to extract the top 100 covariates based on both Pearson and Spearman correlations (Figure 4, Step 10). Approximately 20 of the top 50 covariates in these two lists were already known to be associated with blinding diseases in humans, including *Aipl1, Cabp4, Cnga1, Crx, Gnat1, Gngt1, Guca1a, Guca1b, Kcnv2, Nr2e3, Nrl, Pcdh21, Ped6b, Prcd1, Rcvrn, Rdh12, Rgs9bp, Rom1, Rp1,* and *Rs1*. For example, *Rdh12* (*r*=0.97 with *Rho*) has a well characterized association with a Leber congenital amaurosis (*LCA3*). The remaining members in this list of top 100 are even more interesting and are all highly expressed in the eye, but are not yet known to be associated with retinal disease. This set of disease candidates includes *Ankrd33, C2orf71, Slc24a1, Mcf2l2, Reep6, Mak, C11orf48*, and *Wdr17*.

#### Candidates for photoreceptor disease

By combining the list of transcripts that covary with rhodopsin with data on human retinal disease in RetNet, we generated candidates for seven uncloned human retinal diseases (Table 3). This was done by aligning *Rho* gene covariates such as *Wdr17* with corresponding genes and chromosomal locations in humans. For example, in mice *Wdr17* is located on Chr 8. In humans, the orthologous gene is on Chr 4q34, a region in which Hameed et al. mapped the retinitis pigmentosa 29 locus (*RP29*; [58]). The correspondence suggests that *Wdr17* is a strong candidate for the still uncloned *RP29* gene. Using this same process we nominated genes for six other uncloned human retinal diseases and mutations (Table 3).

**Table 3:** Seven new candidate genes for mapped, but uncloned human disease loci

**New candidate gene**	**Disease**	**Human locus**	**Mouse locus Chr @ Mb**	**Mapping reference**
*Gnb1*	Severe retinitis pigmentosa AR	RP32 1p34.3-p13.3	4 @ 154.13	[81]
*Adipor1*	Retinitis pigmentosa AR	AXPC1 1q31-q32	1 @ 134.28	[82]
*Wdr17*	Retinitis pigmentosa AR	RP29 4q32-q34	8 @ 56.186	[58]
*Egflam*	Macular dystrophy AD	MCDR3 5p15.33-p13.1	15 @ 6.994	[83]
*LOC77938*	Age-related macular degeneration	ARMS2 10q26.13	7 @ 132.59	[84]
*MGC31549*	Retinitis pigmentosa AR	RP22 16p12.3-p12.1	7 @ 125.65	[85]
*2810049P21Rik*	Central areolar choroidal dystrophy AD	CACD 17p13	11 @ 68.79	[86]

When we examined the top 100 genes that correlate with the expression of Rhodopsin, 7 transcripts were identified that mapped to loci associated with human retinal diseases. For all 7 of these diseases the loci responsible for the disease were mapped but the genes were not identified (see selected loci on the RetNet Database, see Below). The 7 mouse genes and the diseases associated with the human loci are listed in this table.

Analysis of differences in expression across tissue types has previously been used to generate candidate genes for retinal disease. Lord-Grignon and colleagues used expressed sequence tag (EST) libraries to link *FAM57B* (their EST AW964851) to retinitis pigmentosa 22 (*RP22*) [59]. Data in HEIMED amplified the association between *FAM57B* and *RP22*. The mouse ortholog of *FAM57B*, *1500016O10Rik,* is highly expressed in eye and covaries tightly with other photoreceptor genes such as *Imphd1, Unc119, Camta2, Pitpnm1, Tulp1*, and *Rho* (rank correlation between 0.84 and 0.75, in that order).

#### Genetic covariance of retinal ganglion cell transcripts

We end this section by providing a more complex example that relies on a set of three signature genes associated with retinal ganglion cells taken from Table 2. The analysis that follows can be applied to most of the short lists of primary signature genes in this table for a wide variety of cell types—from corneal squamous epithelium to vitreal hyalocytes.

1. *Thy1*: Thymus antigen 1, probe set 1423135_at, has a 2.7-fold strain variation and a mean expression of 11.1 units in the whole eye. *Thy1* is a classic ganglion cell maker [60,61] that maps to Chr 9 at about 44 Mb.

2. *Gap43*: Growth-associated protein 43 kDa, probe set 1423537_at, has a 4.0-fold strain variation and a mean expression of 9.0 units. *Gap43* is probably only expressed in a subset of retinal ganglion cells. The study by Ivanov and colleagues [61] demonstrated high relative enrichment of *Gap43* mRNA in ganglion cells. The gene maps to Chr 16 at 42 Mb.

3. *Nrn1:* Neuritin 1, probe set 1428393_at, has a 2.0-fold strain variation and a mean expression of 10.6 units. Neuritin 1 has been shown to be a ganglion cell marker in microarray studies of human and mouse retinas [61,62]. This activity-dependent gene has also been linked with hypoxia [63] and glaucoma [64]. It maps to Chr 13 at 37 Mb.

These three signature genes covary well with each other, as well as with *Chrna6*. One reason to select a set of genes is to ensure that results are less sensitive to microarray measurement error and variation in expression of the signature genes and among cell subtypes.

We can use these three signature transcripts to generate a synthetic ganglion cell trait (a principal component projection) that represents their common or consensus variability. This synthetic trait can be used as bait to extract other transcripts that covary (Figure 8). As expected, the genes used to assemble the synthetic trait appear in this list (rows 1, 3, and 18). What is more interesting are the other top covariates, including *Cplx1, Nsg1, Snca, Nptxr, Kif5a, Stmn2, Atp2b2, Chst1, Psck2, Rab33a, Syn2, Nef3, Nrip3,* and *Nsg2*. Three of these genes are already known to be expressed relatively selectively in retinal ganglion cells (*Cplx1, Nef3,* and *Stmn2* [61,65]). The others are either candidate markers for retinal ganglion cells or are genes expressed in other cell types that covary with ganglion cells.

#### Correlations between expression and cell number

The correlation between numbers of ganglion cells and the synthetic expression trait is 0.41 (n=27, p=0.04, trait 10650). While this number is significant, it indicates that 80% of the variability in gene expression is unrelated to total numbers of these neurons. The correlations between individual transcripts listed in Figure 8 and numbers of retinal ganglion cells (trait 10650) range from a low of 0.16 for *Nef3* to a high of 0.53 for *Chst1*. For example, *Chrna6* has a very weak correlation of 0.18. Natural variation in *Chrna6* levels among strains could be influenced by gene expression in amacrine cells. This could contribute to the high correlation of *Chrna6* with *Gria4*, the GLUR4 AMPA receptor that is expressed heavily in AII amacrine cells.

Can we discover better markers that are more tightly linked to ganglion cell number? One approach is to reverse the process and start with the number of cells. As mentioned previously, *Fbxl20* is one of several genes that covaries tightly with ganglion cell number (r>0.70), and this gene maps to Chr 11 at 98 Mb, the location of the *Nnc1* locus that is known to control variation in retinal ganglion cell numbers in the BXD strains [15]. While the statistics are compelling, *Fbxl20* has comparatively low expression in the adult eye. Furthermore, its expression is also low throughout retinal development [66]; see also Mouse Retina SAGE Library database. *Fbxl20* is closely linked with several strong *Nnc1* candidate genes including *Crkrs* (1438831_at), *Casc3* (1441274_at), and *Thra* [15]. These candidates have higher expression and also covary with ganglion cell number. We conclude that *Fbxl20* is likely to be a genetic marker for ganglion cell number due to its chromosomal linkage to *Nnc1*, not because of a functional or developmental connection (for more details on linkage disequilibrium and their analysis using partial correlations, see [41].

#### Constructing coexpression networks

**Figure 9:**
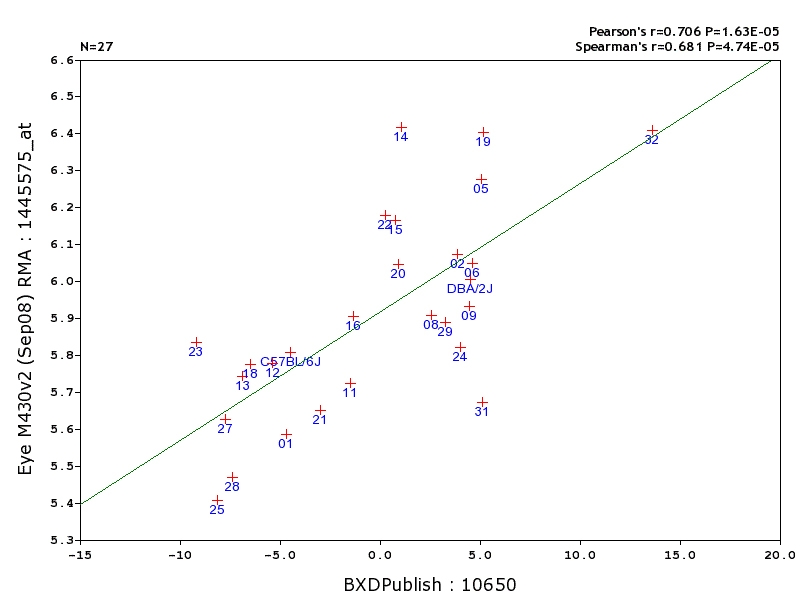
Retinal ganglion cells correlation with *Fbxl20*. x-axis units are in 1000s relative to the mean value of about 58,000 cells. y-axis units are log2 signal intensity. *Fbxl20* is physically linked to the *Nnc1* locus on Chr 11. Despite the strong genetic and statistical association, this gene is unlikely to cause variation in cell number (see text). The effect is likely to be due to linkage disequilibrium. How to generate a correlation graph between a probe set and a phenotype: Step 1. With the descriptors set as “Choose Species=Mouse, Group=BXD, Type=Eye mRNA, Database=Eye M430v2 (Sep08) RMA,” search the ANY search box for the gene *Fbxl20* (1445575_at). Place a check in the box by the 1445575_at probe and click the Add to Collection button. Step 2. Return to the search page and change “Type” to Phenotypes, and “Database” to BXD Published Phenotypes. In the ANY box, search “Retinal Ganglion Cell Number” or 10650. Place a check in the box next to “recordID/10650 – Retinal Ganglion cell number” and click '**Add to Collection**' button. Step 3. Follow the instructions from Figure 6 to arrive at the correlation scatter plot shown.

**Figure 10:**
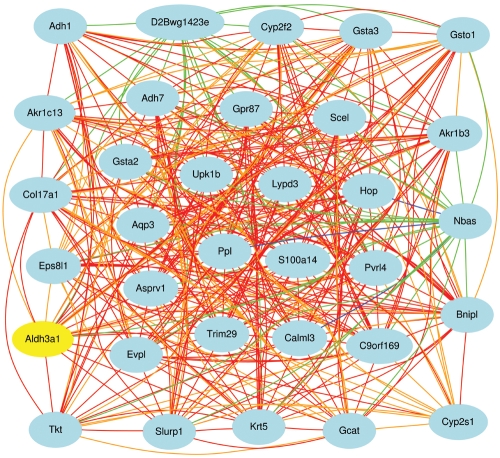
Expression network for cornea. All transcripts connected by red and orange lines covary with each other with positive genetic correlations above 0.7 and between 0.5 and 0.7, respectively. Blue and green lines are the corresponding negative correlations. How to generate this figure: Step 1. Follow the steps in the legend to Figure 8 to generate a correlation of transcripts, in this case using signatures from Table 2 such as *Aldh3a1*. Step 2. Select no more than 100 of these transcripts using the check boxes to the left of each transcript or trait and use the '**Add to Collection**' button to move the selected traits into your Trait Collection window. Step 3. Click on the 'Network Graph' button. Step 4. Adjust the control parameters of the graph.

A list of transcripts such as that in Figure 8 is generated by comparison to a single trait (Figure 9), but there are good reasons for examining larger networks. For example, in Figure 10 we have generated a matrix of correlations among genes with high coexpression with Aldh3a1 and other transcripts with comparatively high expression and selectively expressed in the cornea. Connections between transcripts are defined by genetic correlations computed using strain means. The correlation structure among members of this network is caused almost entirely by a set of sequence differences among strains that generate consistent differences in steady-state mRNA levels among adult strains of mice in the eye and cornea. One of the advantages of using the BXD family is that we can track down these variants. For example, this corneal coexpression network is influenced strongly by loci on several chromosomes.

#### Pros and cons of mRNA analysis of a complex tissue such as the whole eye

A criticism of data sets generated using a tissue as complex as the whole eye is that cellular heterogeneity makes it difficult to correctly interpret and exploit differences in expression. Variation will often be due to both differences in ratios of cell types and to intrinsic differences in expression within single cell types. As we have demonstrated, there are powerful analytic, genetic, and statistical ways to dissect signals originating from tissues and even single cell types. Only a global expression analysis of the whole eye leaves networks intact in an essentially natural state. These data also provide a normative framework for more refined analysis of single tissues and cell types. Rhodopsin is a classic example, but the lists of signatures for cells and molecular systems in Table 2 can be used to begin studies of networks that can extend from correlation to causation using the QTL mapping methods described in Parts 3 and 4.

### Part 3: Genetic analysis and QTL mapping

**Figure 11:**
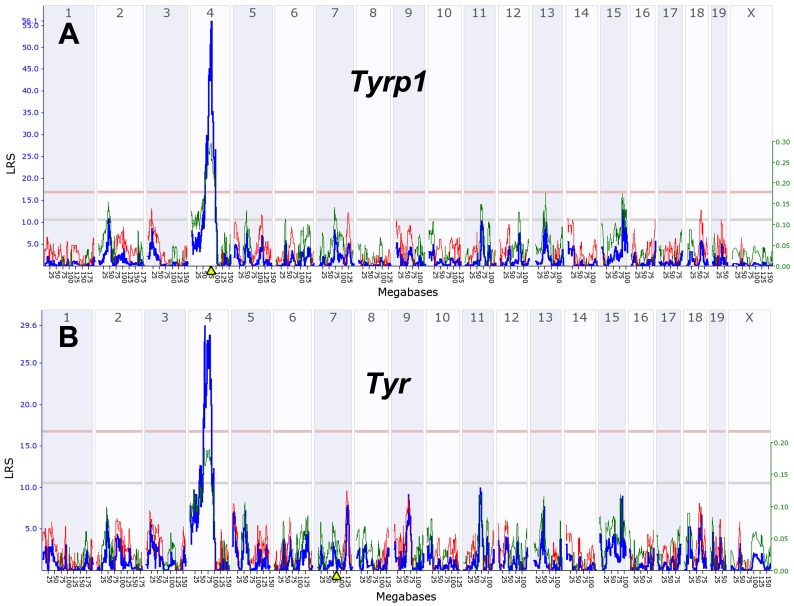
Genetic linkage maps of *Tyrp1* and *Tyr*. **A**: *Tyrp1* expression is controlled by a cis QTL located on Chr 4 at 80 Mb. This location corresponds to the location of the *Tyrp1* itself (triangle on x-axis and the LRS of 57) on Chr 4 at 80 Mb. **B**: A similar map for *Tyr*, a gene that is located on Chr 7 but that has a strong trans-acting QTL. The numbers along the top of each plot represent chromosomes. The y-axis and the bold blue function provides the likelihood ratio statistic (LRS=4.6 x LOD). The two horizontal lines across these plots mark genome significance thresholds at p<0.05 (genome-wide significant, red line) and suggestive threshold (p<0.63, gray line). The thin red and green functions summarize the average additive effects of *D* and *B* alleles among all BXD strains at particular markers. If BXD strains with a *D* allele have higher values than those with a *B* allele at a particular marker then the line is colored green. In contrast, if strains with the *B* allele have higher mean values, the line is colored red. This additive effect size is measure in log2 units per allele. In other words, an additive effect of 0.5 signifies a twofold difference in expression level between strains with *BB* and *DD* genotypes at a marker (log 2 raised to the power of 2×0.5). How to generate QTL maps: Step 1. Link to expression data for a gene of interest using steps in Figure 1. For example, enter the search term “*Tyrp1*” in the ANY field and click the '**Search**' button. Step 2. Click on *Tyrp1* in the Search Results (probe set 1415862_at) to generate the Trait Data and Analysis Form (Figure 4). Step 3. Select the '**Interval Mapping**' button in this form (Figure 4). This will initiate the analysis and display the whole-genome interval map for *Tyrp1*. The steps can be repeated with *Tyr* (1417717_a_at) to generate (panel **B**). You can now zoom in on a single chromosome (e.g., Chr 4) by clicking on the chromosome numbers along at the top of the plot. You can also customize the scale and features of the plot by entering appropriate parameters in the control box.

Heritable variation in mRNA level among BXD strains (Part 1) can be exploited to map well delimited chromosomal regions, or QTLs, that are responsible for differences in gene expression (Figure 11) [40,41,67-69]. The techniques needed to find these QTLs are an integral part of GeneNetwork, and rely on the same software that has been used to map genes that control variation in eye weight, ganglion cell number, lateral geniculate nucleus volume, and differences in ocular dominance plasticity among BXD strains [9,15-17]. The twofold increase in the number of strains relative to almost all previous work using the BXDs (n=68 strains) significantly improves the resolution of QTL maps. The highest possible resolution is about 500 Kb [41], a region that will contain an average of five protein-coding genes, assuming a total of 26,000 genes and a 2,600 Mb genome. A typical QTL mapped using the HEIMED set of 68 BXD strains will often have a confidence interval of 5 Mb and include approximately 50 genes.

#### Variation in mRNAs expression as a micro-trait

The main conceptual difference between mapping standard phenotypes, such as retinal ganglion cell number and mapping mRNA microphenotypes, is that mRNA molecules are directly associated with single genes that have precisely defined single chromosomal locations (see orange triangles along the x-axis in Figure 11A). This makes it possible to break the mapping procedure into two procedures that test two independent hypotheses. The first hypothesis asks whether strain differences in the expression of a gene are controlled by sequence differences in that gene itself (Figure 11A). In other words, is there evidence of genetic self-control? This specific question does not involve screening the entire genome. To answer the question we only need to estimate the statistical linkage between expression of the gene (high, low, or intermediate) and the genotypes of a SNP or microsatellite that is located close to the parent gene. For example, the map of variation in *Tyrp1* expression in Figure 11A has a peak that is aligned precisely with a marker that is located very near to the gene itself. An LRS linkage score of 57 is equivalent to a point-wise (single test) p value of about 4.0×10^−13^, and demonstrates that a genetic polymorphism in or very close to *Tyrp1* influences the level of *Tyrp1* message. A polymorphism in the promoter, enhancer, or a missense mutation that affects RNA stability is a reasonable candidate.

The second hypothesis asks whether variation in the expression of a gene is controlled by sequence differences anywhere else in the genome. The search space now encompasses all regions of all chromosomes. Mapping can only be accomplished by using a set of thousands of markers that have been typed in the BXD strains. For example, the map of *Tyr* expression in Figure 11B has a strong peak, but not on the same chromosome as the *Tyr* gene itself. The peak is on Chr 4 and is actually close to the location of *Tyrp1*.

These two mechanisms are associated with two types of QTLs that are unique to gene expression studies. QTL peaks that overlap the immediate neighborhood of the parent gene are called cis-acting expression QTLs, or cis QTLs for short. *Tyrp1* in Figure 11A is a good example. In contrast, transcripts and their probe sets, such as *Tyr* in Figure 11B, have QTLs that map far from the parent gene itself—usually on a different chromosome. These QTLs are called trans-acting expression QTLs, or trans QTLs. A single transcript can have only one correct cis QTL. In contrast, a single transcript can have several trans QTLs—in some cases three or more. A single gene can produce multiple transcripts and sequence fragments, and each of these can be associated with its own pattern of cis and trans QTLs. For example, the multiple probe sets of *Tyr* detect both cis and trans effects, a finding that emphasizes the complexity of mRNA processing and the critical need for high quality annotation of probe sets [70].

#### Cis QTLs

Well over 10% of probe sets in the eye data set are associated with statistically significant cis QTLs with LRS scores greater than 15 (LOD>3.3; see Figure 2D for a typical search string used to find cis QTLs). This confirms the reasonable expectation that genes will often harbor internal sequence variants that modulate their own expression. The false discovery rate among cis QTLs with LRS>15 is low—well under 0.01 [71,72] for the simple reason that mapping a cis QTL involves a single test of linkage between variation in expression and a marker close to the parent gene (usually within 2 Mb). No statistical adjustment is needed for multiple tests. As a result a standard p<0.05, corresponding to an LRS of 6 and a LOD of merely 1.3, is sufficient. Of the six signature genes that we introduced in Part 1 (*Rho, Chrna6, Chat, Gpnmb, Tyrp1, and Aldh3a1*), three have cis QTLs (*Tyrp1, Gpnmb*, and *Chrna6*). *Tyrp1* has a strong cis QTL with an LRS score of 56, whereas *Chrna6* has a more modest cis QTL with an LRS score of 10.2. The higher the score, the more precise the QTL position will be. For example, the *Tyrp1* gene is located on Chr 4 at 80.3 Mb, and its position coincides perfectly with the LRS peak between 78.5 and 80.8 Mb (Figure 11A). At the other extreme, the weak cis QTL for *Chrna6* is much broader and extends from 15 to 45 Mb, centered around the gene’s position at 29 Mb. When a cis QTL is this broad, the likelihood that the sequence variant is within the gene itself is somewhat lower and the distinction between cis and trans begins to be blurred.

#### Trans QTLs

Trans QTLs are also extremely common in the HEIMED. Over 7,000 probe sets are associated with trans QTLs with LRS values greater than 15. One remarkable feature of these QTLs in the eye data set is that 50% map to a single region of the middle of Chr 4 (60 to 90 Mb). For example, the *tyrosinase* gene (*Tyr,* probe set 1417717_a_at, also known as the albino or *c* locus) is located on Chr 7, but its expression is modulated strongly by a QTL on Chr 4 at 80 Mb (Figure 11B, Table 4).

**Table 4:** Cis-trans gene pairs (cis=cause, trans=target).

Target transcript with trans-QTL								Causal candidate gene (cis-QTL)					
Target gene		Exp Level	Affy ID	LRS	QTL Chr	QTL Range	Tissue or function	*Candidate gene*		Affy ID	Chr	Mb	LRS
*Tyr*	*tyrosinase (albino c locus)*	10.7	1417717*	29.6	4	78–81	pigmentation	*Tyrp1*	*tyrosinase related protein 1 (b locus)*	1415862*	4	80.3	56.2
*Dct*	*dopachrome tautomerase*	14	1418028*	20	4	60–95	retinal pigment epithelium	*Tyrp1*	*tyrosinase related protein 1 (b locus)*	1415862*	4	80.3	56.2
*Asip*	*agouti signaling protein*	6.2	1420516*	19.8	1	58–63	pigmentation	*Nbeal1*	*neurobeachin like 1*	1441444*	1	60.2	10
*Mela*	*melanoma antigen*	8.6	1433438*	131	9	74–77.5	retinal pigment epithelium	*Myo5a*	*myosin 5a (dilute d locus)*	1419754*	9	75	159
*Mcam*	*melanoma cell adhesion molecule*	7.6	1416357*	31.4	1	138–143	melanocyte and endothelial cell function	*Nek7*	*NIMA (never in mitosis a)-related expressed kinase 7*	1416816*	1	140.3	19.2
*Bfsp2*	*phakinin*	12.9	1434463*	25.6	3	143–147	lens	*Col24a1*	*procollagen type 24 alpha 1*	1453418*	3	145.5	11.3
*Lenep*	*lens epithelial protein*	11.3	1424423*	25.2	5	111–114	lens	*Cryba4*	*crystallin beta A4*	1420686*	5	112.5	24.1
*Cd59a*	*protectin, Cd59a antigen*	10.8	1418710*	48.5	13	62–69	retina, immune function	*Zfp58*	*zinc finger protein 58*	1455945*	13	68	13
*Ahnak*	*desmoyokin*	7.1	1428058*	56	5	110–114	epithelium, motility	*Cryba4*	*crystallin beta A4*	1420686*	5	112.5	24.1
*Eif4g2*	*translation initiation factor 4, gamma 2*	12.9	1415862*	39.8	4	94–97	photoreceptor function	*Hook1*	*hook 1*	1438018*	4	95.5	18.1
*Cks1b*	*CDC28 protein kinase 1b*	8.6	1448441*	39.1	1	193–195	unknown, not retina	*Rd3*	*retinal degeneration 3*	1452355*	1	193.7	23.6
*Ptp4a1*	*PRL1 cone phosphatase*	11.5	1449322*	42	5	111–115	retinal function	*Kctd10*	*potassium channel tetramerisation domain 10*	1423694*	5	114.6	20
*Ctnna1*	*catenin alpha 1*	12.3	1448149*	20.5	14	9.5–18.5	unknown, possible retinal function	*Atxn7*	*ataxin 7 (photoreceptor degeneration)*	1442186*	14	12.9	48.1
*Pten*	*phosphatase and tensin homolog*	11.6	1422553*	21	5	136–139	unknown, possible retinal function	*Zpf68*	*zinc finger protein 68*	1457462*	5	138.8	64.5
*Ldhb*	*lactate dehydrogenase 2, B chain*	12.4	416183*	17	8	14–17	retinal ganglion cell function	*Dlgap2*	*PSD-95/SAP90-binding protein 2*	1457979*	8	14.9	14
*Nars*	*asparaginyl-tRNA synthetase*	11.9	1452866*	39	1	174–178	neuronal function	*Fmn2*	*formin 2*	1450063*	1	176.7	20.5
*Pacsin3*	*protein kinase C and casein kinase substrate in neurons 3*	10.6	1437834*	34.9	3	144.5–147	neuronal function	*Sh3glb1*	*SH3-domain GRB2-like B1 (endophilin)*	1418012*	3	144.6	22.8
*Matr3*	*matrin 3*	8.9	1436796*	61	11	28–34	cytoskeleton	*Ccdc88a*	*girdin (hook-related protein 1)*	1436025*	11	29.4	17.3
*Ifit1*	*interferon-induced tetratricopeptide repeats 1*	8.7	1450783*	33	2	62–65	ocular immune function	*Ifih1*	*interferon induced with helicase C domain 1*	1426276*	2	62.4	40.3
*Psat1*	*phosphoserine aminotransferase 1*	10.4	1454607*	35.5	8	32.0–37.5	unknown, possible retinal function	*Ppp2cb*	*protein phosphatase 2a, catalytic, beta*	1421823*	8	35	21.6
*Ube2i*	*ubiquitin-conjugating enzyme E2I*	11.3	1422712*	32	18	23.5–34	unknown	*Stard4*	*StAR-related lipid transfer (START) domain containing 4*	1429239*	18	33.3	27.3
*Hspbap1*	*HSPB associated protein 1*	8.1	1441900*	30.7	3	141–146	lens and neuronal function	*Unc5c*	*Unc-5c, netrin 1 receptor*	1449522*	3	141.4	10
*Myoc*	*myocilin*	10.8	1450468*	21.8	2	35–41	glaucoma	*Gsn*	*gelsolin*	1456312*	2	35.1	None
*Ninj1*	*ninjurin 1*	11.6	1438928*	34.4	17	31–39	translation, oxidative stress	*Cryaa*	*crystallin, alpha A*	1452486*	17	31.4	None
*Epgn*	*epithelial mitogen*	8	1449994*	36.1	14	99–105	ocular vasculature and pigmented cells	*Ednrb*	*endothelin receptor type B (Hirschsprung disease type 2)*	1437347*	14	102.7	5

* Affymetrix probe set IDs have been truncated to the unique id number. Search GeneNetwork using the asterisk.

#### Low-hanging fruit: Cis-Trans QTLs pairs

Genes associated with cis QTLs control their own expression. They may also control the expression of other genes that are part of the same molecular networks, particularly those with significant trans QTLs. There are numerous examples of genes with trans QTLs that can be linked to a small number of candidate genes with strong cis QTLs (Table 4). The *Tyr* (albino locus) and *Tyrp1* (the brown locus) are perhaps the best example of such a pairing. The *Tyrp1* gene of DBA/2J contains two missense mutations that contribute to the brown pigmentation phenotype (Cys110Tyr and Cys326Tyr) [73]. In addition to these two mutations, *Typr1* expression is associated with a massive cis QTL in which the *D* haplotype has 25% higher mRNA expression than the *B* haplotype (Figure 11A). Thus, sequence variants in and around *Tyrp1* of the two parental strains and all of the BXD strains affect mRNA levels for a wide variety of transcripts.

**Figure 12:**
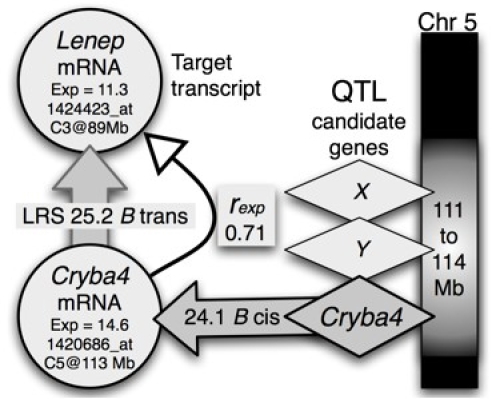
Model of gene expression. Data in this figure are taken from Table 4. *Cryba4* is the most compelling of several candidates on Chr 5. Exp: mean expression level. Positions of genes are abbreviated C3@89Mb=chromosome 3 at 89 Mb. The correlation between expression of transcripts is indicated by the curved arrow (r=0.71). The large vertical arrow between *Cryba4* and *Lenep* mRNAs is a causal hypothesis that requires testing. It is also possible that this arrow originates from one of the other candidate genes.

There are numerous other revealing examples of cis-trans QTL pairs. Expression of the lens epithelial protein (*Lenep*) is strongly affected by a trans QTL on Chr 5 between 111 and 114 Mb. *Cryba4* is the most compelling candidate gene in this interval and is itself associated with a large cis QTL. We have assembled a table that includes 24 other intriguing and possibly functional cis-trans pairs (Table 4). In each case, the general model is similar to that in Figure 12, with a candidate gene shown to the right of Table 2 and the target transcript with its trans QTL shown to the left. In many cases, a trans QTL will not be associated with a known cis QTL. Sequence variants that affect expression may instead alter protein sequence. We have included two examples of this type in Table 4. Myocilin and ninjurin both have strong candidate genes—gelsolin (*Gsn*) and crystallin alpha A (*Cryaa*), respectively, and neither has a cis QTL.

For each of the QTL intervals, there are often three or more candidate genes (e.g., *Cyrba4*, genes labeled gene X and gene Y in Figure 12). It is therefore necessary to evaluate the relative merits of candidates. Important factors include the following:

1. The locations of candidate genes relative to the peak QTL linkage score

2. Locations of candidate genes relative to the density of sequence variants that are effective (segregating) in the BXD family

3. Expression levels of candidate genes in the adult eye

4. The strength of cis QTLs and the functional significance of coding variants in candidate genes

5. Genetic correlations between the expression of candidate genes and the target transcript (the gene being mapped)

6. Known functions, interactions, and molecular pathways of candidate genes relative to the target gene

7. Correlations of expression between candidate genes and the target gene across multiple tissue types (tissue correlations)

These factors can be used as part of a protocol to rank candidates for in-depth molecular and functional assays. For example, *Ldhb* expression (row 15 in Table 4) may be affected either by *Arhgef10*, a Rho guanine nucleotide exchange factor, or by *Dlgap2*, also known as postsynaptic PSD-95/SAP90-binding protein 2. *Dlgap2* is favored because its expression is highly correlated with that of other ganglion cell genes. This gene is listed as a candidate in Table 4. However, *Arhgef20* is favored because it has a twofold higher expression in eye and is associated with a stronger cis QTL.

#### Variability among probe sets for single genes

Genes often give rise to multiple transcripts. The expression patterns of these isoforms and differences in their processing will often vary among strains and among cell types. As a result, different probe sets for single genes can have different sets of QTLs. For example, the expression level of tyrosinase mRNA is estimated by three probe sets that target progressively more distal regions of the transcript. Expression estimates generated using the most proximal 5′ probe set (1417717_a_at) indicate that the mRNA is modulated by a trans QTL on Chr 4. In contrast, the most distal probe set (1456095_at) is modulated strongly by a cis QTL with an LRS of 50.4. A probe set with an intermediate position (1448821_at) is modulated by both trans and cis QTLs. This emphasizes that expression of different exons, splice variants, and parts of the 3′ UTR can be controlled by different sets of QTLs. MicroRNAs are one interesting source of these differences. They typically target motifs in the 3′ UTR and modulate translational efficiency and mRNA catabolism [74]. Strain-specific sequence variants in the 3′ UTR sequence will also have important effects on the final position of the poly-A tail and on rate of message degradation.

### Part 4: Genomic networks and complex analysis of expression

#### Whole eye genetic transcriptome analysis

**Figure 13:**
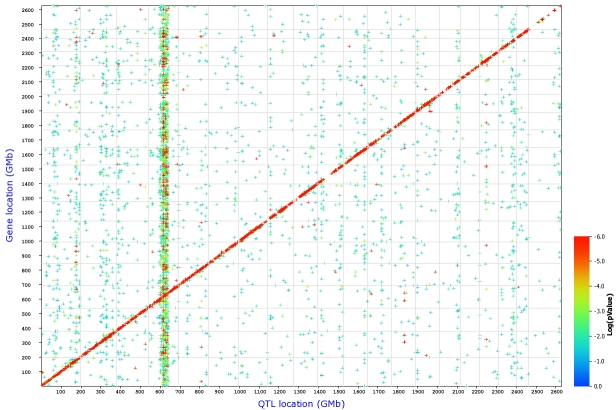
Genome-wide distribution of QTLs. Each point represents a single probe set. The x-axis gives the position of the QTLs (the single best QTL for those probe sets at a false discovery rate of 0.2), whereas the y-axis gives the position of the gene or probe set target itself. Positions are measured in genome-wide Mb (GMb) from Chr 1 through to the Chr Y (2600 GMb). The gray lines mark chromosome boundaries, and the significance level of individual QTLs are color-coded. High LRS values (low genome-wide P values) are represented by red, intermediate LRS values by green, and low values by blue. A large number of highly significant cis QTLs form a diagonal (red) line. Vertical bands such as that at 610 GMb (Chr 4 at 80 Mb) represent groups of transcripts that have trans QTLs at the same location. The major trans-acting band at 610 GMb corresponds to the *Tyrp1* locus. How to perform a genome-wide scan by examining all of the QTLs in the HEIMED: Step 1. Link to GeneNetwork and select GenomeGraph from the “Search” pull-down menu at the top left of the page. Step 2. Configure the pull-down menus to read “Choose Species=Mouse, Group=BXD, Type=Eye mRNA, Database=Eye M430v2 (Sep08) RMA.” Step 3. Select the '**Mapping**' button. This will generate the Whole Transcriptome Mapping page. You may adjust the false discovery rate (FDR). In our studies, we chose an FDR of 0.2. The entire data set of values used to construct this type of graph can be downloaded at GeneNetwork.

**Figure 14:**
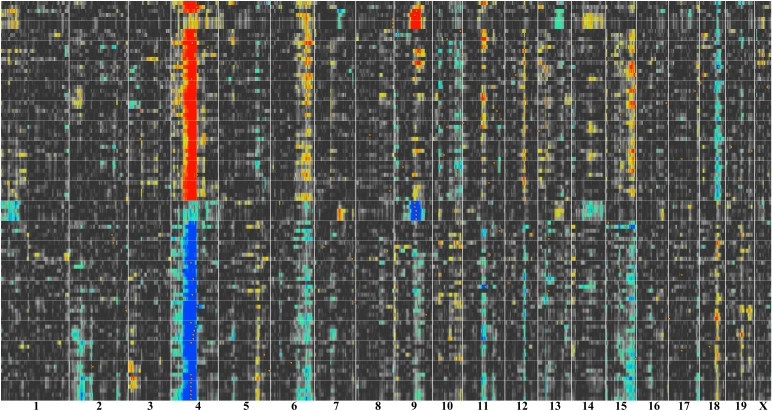
QTL cluster map for coat color in the BXD RI strains. Chromosomes are listed along the bottom of the figure from 1 to X. Each row corresponds to a QTL map for a single transcript. The intense red and blue bands on Chr 4 correspond to significant QTLs on Chr 4 centered at approximately 80 Mb—the location of *Tyrp1*. The lower blue section of this Chr 4 band corresponds to transcripts whose expression is higher in strains with a *B* haplotype on Chr 4, whereas the upper red section corresponds to transcripts whose expression is higher in strains with the mutant *D* haplotype. In addition, there are distinct but less intense bands on Chrs 6, 9, 15, and 18. How to extract data based upon phenotype using “Coat Color” to determine the possibility that the Chr 4 trans-acting band is related to pigmentation: Step 1. Open either the main website GeneNetwork. Step 2. Set up the Find Records field to read “Choose Species=Mouse, Group=BXD, Type=Phenotypes, Database=BXD Published Phenotypes. Step 3. Enter the search term “Coat Color” in the ANY field and click on the '**Search**' button. Step 4. Select RecordID/11280-Coat Color to generate the Trait Data and Analysis page. Step 6. In the Analysis Tools section, locate the options for Trait Correlations. There are several options in this area: Choose Database, Calculate, and Return. Under Choose Database select the Eye M430V2 (Sep 08) RMA database, under Return select top 200, and finally select '**Trait Correlations**'. The Correlation Table is constructed listing the top 200 correlates that are associated with the eye and coat color. Step 7. Click on a limit of 100 of the highest correlates, making sure that you include genes that are known to be associated with coat color and the eye. After 100 probe sets are chosen, select the '**QTL Heat Map**' function.

The analysis of cis and trans QTLs can be scaled up to the level of the entire transcriptome. One way to display global genetic control of transcription is to plot chromosomal positions associated with the highest LRS scores for transcripts against the locations of their parent genes. Transcripts that are controlled by local sequence variants—cis QTLs—will map along a diagonal (Figure 13). In contrast, trans QTLs, such as those listed in Table 4, map as vertical clusters. A prominent feature of these graphs is the presence of a small number of chromosomal hot spots, or QTL hubs, that modulate expression of large numbers of transcripts distributed across the entire genome [41,75,76]. A QTL hub will influence the expression of many more genes than expected from the known distribution of genes across the genome. For example, sequence variants on distal chromosome 1, probably associated with formin 2 (*Fmn2*), modulate many of the amino-acid tRNA synthetase genes that are critical in protein translation in neurons [41]. By far the most impressive single source of variation in gene expression in the eyes of BXD strains is generated by a major QTL hub on Chr 4 centered on *Tyrp1* (Figure 13, Figure 14).

#### Chromosome 4 and *Tyrp1*

In the entire transcriptome data set, a total of 7109 probe sets have a trans QTL with an LRS score above 15. Nearly 10% of these are controlled by a QTL between 77 and 83 Mb on Chr 4 (n=605, using this search text in the ALL field: “transLRS=(15 30000 10) LRS=(15 30000 Chr4 77 83)”). This is a remarkable enrichment considering that this 6 Mb interval makes up only 0.23% of the genome and contains only 16 protein-coding genes. Six genes in this region have transcripts with cis QTLs above 15, including *Tyrp1*, *C9orf150, Nfib, Ttc39b, Snapc3*, and *4930473A06Rik*. All of these are positional candidates, but only *Tyrp1* is a compelling biologic candidate. *Tyrp1* is mutated in DBA/2J and in BXD strains with the *D* haplotype. The *Tyrp1* brown mutation is one of two mutations that contributes significantly to pigment dispersion glaucoma in DBA/2J [77]. As expected for an eye-specific pigmentation effect, the Chr 4 QTL hub is not present in other tissues that have been studied in BXD strain, such as brain, liver, or hematopoietic stem cells.

**Figure 15:**
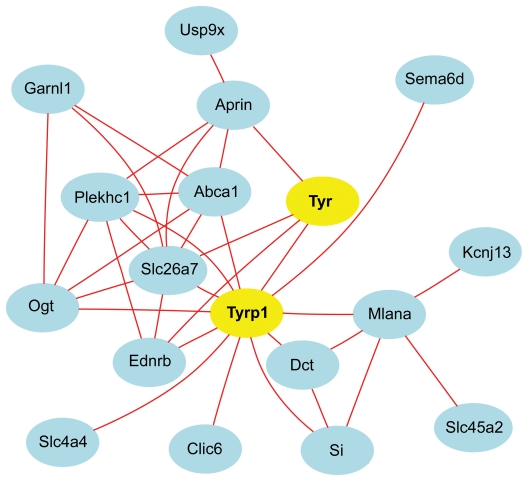
Network graph makes highlights transcripts associated with *Tyrp1* and *Tyr*. The 18 transcripts (nodes) in the graph are connected by Pearson correlation coefficients greater than 0.7 (red lines). How to define genetic networks in the eye: Step 1. Open either the main website GeneNetwork. Step 2. Set up the Find Records field to read “Choose Species=Mouse, Group=BXD, Type=Eye mRNA, Database=Eye M430v2 (Sep08) RMA. Step 3. Enter the search term “*Tyrp1*” in the ANY field and click on the '**Search**' button. Step 4. Select ProbeSet/1415862_at to generate the Trait Data and Analysis form. Step 6. In the Analysis Tools section, locate the options for Trait Correlations. Under Choose Database select the Eye M430V2 (Sep 08) RMA database, under Return select top 100, and select '**Trait Correlations**'. A Correlation Table is constructed listing the top 100 correlates associated with the *Tyrp1* expression variation in the eye. Step 7. Click on as many as 100 of the correlates. For the graph above, we have specifically selected the first *Tyrp1* probe set and the next 17 probe sets of the 100 genes that are of the most interest and highest correlation. After the probe sets are chosen, select the '**Add to Collection**' function. Step 8. At the BXD Trait Collection page, select all or the genes of interest and select the '**Network Graph**' function. For this figure an absolute value of 0.7 was set as the correlation threshold in the user defined settings. The network is drawn using certain default parameters that can easily be changed. The network displays are interactive and allow the user to link to interesting nodes and traits for further analysis.

A list of the top correlates of *Tyrp1* include many well known pigment-associated genes, such as *Dct, Slc45a2, Slc26a7, Tyr, Usp9x,* and *Abca1*. Transcripts of most of these genes map to Chr 4 close to the location of *Tyrp1*. It is highly likely that either the well known coding mutations in *Tyrp1* [73] or significant strain variation in the expression of this gene generate widespread direct and indirect effect on the expression of hundreds of genes across the genome (Figure 15).

#### The modulation of genomic signatures

In part 3, we illustrated how pairs of genes controlled by cis and trans-acting QTLs (Table 4) can be used to assemble models of gene-gene interactions such as those highlighted in Figure 13. This kind of analysis can be extended to large sets of transcripts and groups of QTLs using unique tools that are built into GeneNetwork.

**Figure 16:**
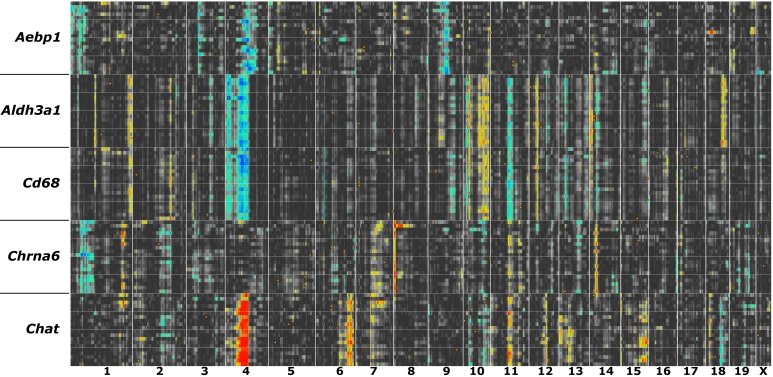
QTL signatures for cells and tissue types. Five cohorts of transcripts (n=20) were generated using signature genes listed in Table 2: *Aebp1* (sclera), *Aldh3a1* (cornea), *Cd68* (anterior segment and macrophages), *Chrna6* (retinal ganglion cells), and *Chat* (starburst amacrine cells). Each row in this figure is color-coded by the strength and polarity of genetic control. Chromosome regions that exert strong control are either blue (*B* alleles contribute to higher expression) or red (*D* alleles contribute to higher expression). Each tissue type has one or more chromosomal regions with relatively consistent QTL peaks. In contrast, the strong modulation by a QTL near *Tyrp1* on Chr 4 is a notable feature across several cell and tissue types. How to identify QTL networks modulating tissue specific gene expression: Step1. Follow the steps in the legend of Figure 8 to generate a set of 10 of more transcripts that covary with signature transcripts listed in Table 2. Step 2. Place the transcript data sets into your BXD Trait Collection (maximum is approximately 100). Step 3. Select traits in your BXD Collection and click on the '**QTL Heat Map**' button.

The expression profiles of a cell or tissue type are the result of unique sets of regulatory elements in those cells or tissues. Loci modulating expression in tissues and cells can be identified using signatures transcripts (Table 2). Probe sets that covary well with signature transcripts can be loaded into the QTL Heat Map function as shown in Figure 16. Heat maps of this type display QTLs for large numbers of transcripts (up to 100, listed to the left) across the entire genome. In Figure 16 we illustrated examples of QTL maps for five different ocular signatures: *Aebp1* (sclera), *Aldh3a1* (cornea), *Cd68* (macrophages), *Chrna6* (retinal ganglion cells), and *Chat* (starburst amacrine cells). The intensity of colors indicates the strength of the QTLs.

#### The signature loci differ between cell types or tissue types

Each of the five tissue types in Figure 16 has a unique pattern of modulatory loci that provide a whole-genome QTL signature. These QTL signatures are distinct. For example, the five scleral signature genes listed in Table 2 (*Aebp1, Bgn, Fmod, Mxra8, Pcolce)* and 15 of their tightest covariates are jointly modulated by QTLs on Chr 3 at 52–58 Mb and Chr 9 near *Col12a1* at 80 Mb. Several cohorts of transcripts have a common band on the distal half of Chr 4 that is most likely due to direct and indirect effects of the *Tyrp1* mutation. The corneal signature (*Aldh3a1*) and the macrophage signature (*Cd68*) have a common band on distal Chr 1. Many other bands are unique to single sets of transcripts. For example, the *Chrna6*-related cohort of transcripts (ganglion cell associated) has unique bands on Chr 1 and proximal Chr 8. As a control, several series of genes were selected that were not correlated and no consistent signature loci were observed.

**Figure 17:**
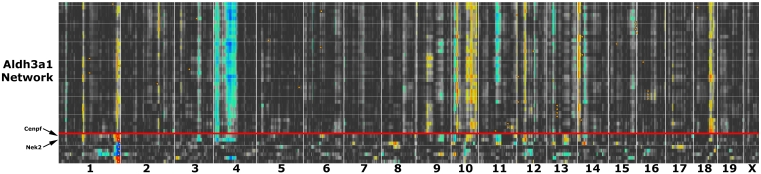
Corneal QTL networks identified using variation in *Aldh3a1* expression level. Notice the distinct vertical QTL bands shared by nearly all transcripts. Chromosomes are numbered at the bottom. Above the red line are transcripts that correlate with the expression of *Adh3a1*. Below the red line are transcripts and genes associated with strong cis QTLs that may cause the transband on distal Chr 1. These genes include *Cenpf, Nek2, Slc30a1, Traf5, Rcor3, Kcnh1,* and *G0s2*. Notice that two of these transcripts (*Cenpf* and *Nek2*) have the same banding pattern as well known corneal signature genes (above the red line). *Cenpf* and *Nek2* are therefore particularly good candidates that may control expression of the corneal network. How to examine a region of a chromosome (Chr 1) to define candidate genes: '**The Advanced Search**' function as described in Figure 2 illustrates complex searches that can be used to find probe sets with low expression, but good signal-to-noise ratio. The Advanced Search query is a string of search parameters that first limits the search to genes with a significant QTL. The first command entered into the ANY box is LRS=(20 200). This limits the search to genes with LRS scores between 20 and 200; thus search results identified 6,451 probe sets. When you run this search an error message occurs indicating that you have generated a list of over 2,000 genes and request that you modify the search to generate a list of fewer than 2,000 genes. Ignore this message and modify the search to limit it to genes within the immediate vicinity of the peak LSR score. The next command cisLRS=(20 200 20) will limit the search to significant LRS scores where the gene lies within 20 Mb of the peak LRS score. Within the HEIMED there are 4,580 probe sets that meet this criterion. The results of this search also generate an error because over 2,000 probe sets were found. Finally, the last command limits the genomic region to distal Chr 1 using Mb=(Chr1 190 200). This search produces 117 probe sets. Finally, if we combine all these search criteria as LRS=(20 200) CisLRS=(20 200 20) mb=(chr1 190 200), the result is 18 probe sets. These candidate genes include *Cenpf, Nek2, Slc30a1, Traf5, Rcor3, Rd3, Kcnh1*, and G0s2. Select these genes by clicking on the box next to their probe set and select '**Add to Collection**'. At the BXD Trait Collection page you will find a list of the genes’ probe sets. Click the '**Select All**' button and then select the '**QTL Heat Map**' function. The QTL Heat Map will compute two transcripts, *Cenpf* and *Nek2*, which have a similar QTL signature as *Aldh3a1*.

When we examine the structure of the regulatory loci for the transcripts that make up this cohort we observe that it is different from most of the other signatures illustrated in Figure 17. Genes associated with strong cis-acting QTLs are prime candidates for upstream modulators of the gene network. *Aldh3a1* has a network [39,78] that includes many of other genes characteristic of the cornea, among them keratin 12, members of the KLF transcription factor family, and the corneal crystalline, *Tkt*. There is a distinct trans-acting band on distal Chr 1, two bands on Chr 4, a broad band on chromosome 10, and a tight band on distal Chr 18. This signature of regulatory loci reflects the unique series of loci that modulate the genetic network controlling the selected expression of genes in the cornea (Figure 17). We can identify the cis-acting elements for the tight trans-acting band on distal Chr 1 using the advance search feature in GN. This allows us to extract candidate genes associated with strong cis QTLs in this band (see legend for steps). Among them are *Cenpf, Nek2, Slc30a1, Rd3, Traf5, Rocr3, Kcnh1*, and *G0s2*. Since two of these (*Cenpf* and *Nek2*) have the same genome-wide QTL pattern as well known corneal signature genes, these genes are good candidates for upstream modulators of the corneal network.

### Conclusion

We have provided a large resource that is especially useful for characterizing molecular and genetic networks in the eye and for tracking down sequence variants related to differences in expression and disease susceptibility. By treating changes in mRNA levels as a phenotype, differences in transcriptional control can be evaluated using traditional QTL mapping methods. Variation at the transcript level can be correlated with other higher order transcriptional networks, as well as with cellular and morphological differences. This approach can be used to define molecular signatures within tissues and cells of the eye, identify candidate genes for human ocular disease, assemble genetic networks regulating tissue specific gene expression, and identify complex interactions among gene variants that generate variation in eye structure and function.

### What comes next

Two technologies will soon greatly enrich the analysis of transcriptional circuitry in eye and other cells and tissues. The first of these is massively parallel sequencing technology. It is now practical to generate up to 600 million independent 50-mer mRNA sequence reads in less than a week from an mRNA sample. The precision and dynamic range of sequence-based estimates of expression level will be much improved compared to array-based hybridization methods. It will be practical to quantify mRNA isoforms without the biases of probe sequence selection and hybridization reactions. The second innovation is cell-specific RNA profiling methods [79] that make it practical to generate comparatively accurate expression data for individual cell types in genetically engineered lines of mice. We can soon expect far more comprehensive and specific lists of genes for several important cell and tissue types that can be used to assemble multicellular expression networks in eye.

## Appendix 1.

Sex differences in gene expression based on a subset of 93 strains for which matched male and female data are available. To access the data, click or select the words “Appendix 1.” This will initiate the download of a Microsoft Excel (.xls) file that contains the data.
